# Gut Microbial Perturbation and Host Response Induce Redox Pathway Upregulation along the Gut–Liver Axis during Giardiasis in C57BL/6J Mouse Model

**DOI:** 10.3390/ijms24021636

**Published:** 2023-01-13

**Authors:** Avinash V. Karpe, Melanie L. Hutton, Steven J. Mileto, Meagan L. James, Chris Evans, Amol B. Ghodke, Rohan M. Shah, Suzanne S. Metcalfe, Jian-Wei Liu, Tom Walsh, Dena Lyras, Enzo A. Palombo, David J. Beale

**Affiliations:** 1 Environment, Commonwealth Scientific and Industrial Research Organization, Ecosciences Precinct, Dutton Park, QLD 4102, Australia; 2 Department of Chemistry and Biotechnology, School of Science, Computing and Engineering Technologies, Swinburne University of Technology, Hawthorn, VIC 3122, Australia; 3 Infection and Immunity Program, Monash Biomedicine Discovery Institute and Department of Microbiology, Monash University, Clayton, VIC 3168, Australia; 4 Health and Biosecurity, Commonwealth Scientific and Industrial Research Organization, Ecosciences Precinct, Dutton Park, QLD 4102, Australia; 5 Department of Horticulture, Queensland Alliance for Agriculture and Food Innovation, The University of Queensland, St. Lucia, QLD 4072, Australia; 6 Environment, Commonwealth Scientific and Industrial Research Organization, Agricultural and Environmental Sciences Precinct, Acton, Canberra, ACT 2601, Australia

**Keywords:** host–parasite interactions, systems biology, giardiasis, gut–liver axis, integrated multiomics, metabolic pathways

## Abstract

Apicomplexan infections, such as giardiasis and cryptosporidiosis, negatively impact a considerable proportion of human and commercial livestock populations. Despite this, the molecular mechanisms of disease, particularly the effect on the body beyond the gastrointestinal tract, are still poorly understood. To highlight host–parasite–microbiome biochemical interactions, we utilised integrated metabolomics-16S rRNA genomics and metabolomics–proteomics approaches in a C57BL/6J mouse model of giardiasis and compared these to *Cryptosporidium* and uropathogenic *Escherichia coli* (UPEC) infections. Comprehensive samples (faeces, blood, liver, and luminal contents from duodenum, jejunum, ileum, caecum and colon) were collected 10 days post infection and subjected to proteome and metabolome analysis by liquid and gas chromatography–mass spectrometry, respectively. Microbial populations in faeces and luminal washes were examined using 16S rRNA metagenomics. Proteome–metabolome analyses indicated that 12 and 16 key pathways were significantly altered in the gut and liver, respectively, during giardiasis with respect to other infections. Energy pathways including glycolysis and supporting pathways of glyoxylate and dicarboxylate metabolism, and the redox pathway of glutathione metabolism, were upregulated in small intestinal luminal contents and the liver during giardiasis. Metabolomics-16S rRNA genetics integration indicated that populations of three bacterial families—*Autopobiaceae* (Up), *Desulfovibrionaceae* (Up), and *Akkermanasiaceae* (Down)—were most significantly affected across the gut during giardiasis, causing upregulated glycolysis and short-chained fatty acid (SCFA) metabolism. In particular, the perturbed *Akkermanasiaceae* population seemed to cause oxidative stress responses along the gut–liver axis. Overall, the systems biology approach applied in this study highlighted that the effects of host–parasite–microbiome biochemical interactions extended beyond the gut ecosystem to the gut–liver axis. These findings form the first steps in a comprehensive comparison to ascertain the major molecular and biochemical contributors of host–parasite interactions and contribute towards the development of biomarker discovery and precision health solutions for apicomplexan infections.

## 1. Introduction

Giardiasis is a gut infection caused by various members of protists of the genus *Giardia*, most notably *G. lamblia* (also called *G. duodenalis* and *G. intestinalis*) [1]. Although it is prevalent in developing and underdeveloped countries, giardiasis has been shown to affect up to 20% of children (<10 years) globally, and accounted for up to 35.2% of the global water-borne disease outbreaks from 2004–2010 [2]. Similar to other waterborne infections, such as cryptosporidiosis, amebiasis, and *Escherichia coli*, giardiasis spreads primarily through faecal-contaminated water [3]. Additionally, *Giardia* spp. also commonly infect livestock, dogs, cats, and wildlife, making the infection zoonotic in nature [4].

*Giardia* spreads through cyst bodies originating from infected matrices or individuals. The mature cyst consists of two trophozoites. These trophozoites attach to intestinal mucosa, mostly in a localised and non-invasive manner [1], and reproduce by asexual binary fusion in duodenum and jejunum within the small intestine [5]. Similar to related protists, *Giardia* is an obligate parasite, and lacks numerous metabolic/genomic systems; its life cycle is highly specialised, and consists of complex growth stages, making its study difficult [6]. The absence of amino acid, nucleic acid, and fatty acid synthesis mechanisms makes in vitro growth of *Giardia* difficult, requiring animal models such as mice, rats, and guinea pigs to study giardiasis [6,7].

Clinical studies have shown increased prevalence of arthritis [8], post-infection irritable bowel syndrome (IBS), allergies, impaired cognition, and myopathy [9] in *Giardia*-infected patients. It is possible that this may be caused by nutrient malabsorption caused during giardiasis, not only in the intestinal tract, but also affecting the biochemical and physiological conditions in other host systems. Recent studies indicate giardiasis causes gut microbiome dysbiosis [10,11,12], particularly a depletion of mucosal anaerobic *Firmicutes* and *Melainabacteria* in mouse hindgut [12], but exactly how this occurs remains unclear.

In light of this, omics platforms such as metagenomics, metaproteomics, and metabolomics have immense potential towards understanding these interactions. Such platforms have been increasingly utilised over recent years to understand the biochemical mechanisms of infections [13,14,15], disorders such as IBS [16,17,18], and complex interactions within environmental samples [19,20]. Thus, the increased sensitivity and discriminatory power of proteomics and metabolomics, coupled with genomic sequencing and robust databases, has the capability to provide a true understanding of the biochemical interactions within the gut during infection [21]. Furthermore, these platforms can provide information of not only the consequences of infection extending beyond the gut, but also, differentiate between various infections and disorders (e.g., giardiasis, cryptosporidiosis, or IBS).

Herein, we investigated the differential metabolic interactomics of giardiasis with respect to other protist (cryptosporidiosis) and prokaryotic (uropathogenic *E. coli* (UPEC)) infections using integrated multi-omics platforms. We investigated numerous tissues and gut luminal washes after infection with *G. lamblia*, *Cryptosporidium parvum*, and UPEC, and applied microbiome 16S rRNA gene sequencing to formulate hypotheses that giardiasis and cryptosporidiosis have diverse effects on the gut microbiome and, thereby, influence metabolic activity in different regions of the gut. Untargeted metabolomics and proteomics revealed how the biochemical interactions between host cells, gut microbiome, and parasite impact metabolism and nutrition-related pathways within the murine gut. This is the first demonstration that different infections produce distinctive alterations in these pathways and that the biochemical effects extend beyond the gut.

## 2. Results

### 2.1. Infection Analysis and Mouse Strain Selection

**Figure 1:**
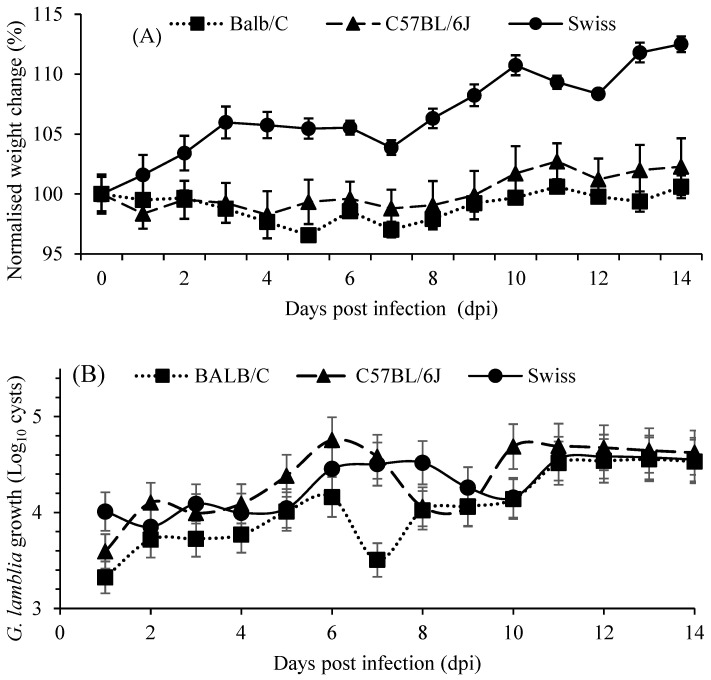
(**A**) Weight, relative to day zero of mice during infection and (**B**) *G. lamblia* colonisation, from faecal shedding, during infection.

Mice from three different genetic backgrounds (Balb/C, C57BL/6J, and Swiss) were tested to identify the optimal strain for *Giardia* infection analysis. During the 14-day study period, the mice did not show visual diarrheic effects. Moreover, visual inspections and weight measurements (Figure 1A) indicated that the health of the mice was not drastically affected. Swiss mice showed considerable weight gain, with an average increase of 3.02 g and, thus, were not deemed appropriate for further study. Infected C57BL/6J and BALB/C mice displayed a slight decrease in weight up to 8 days post infection (dpi). Following this initial weight loss, infected C57BL/6J and BALB/C mice began to regain weight, with an average increase of 0.45 g and 0.11 g, respectively, by the completion of the pilot study. C57BL/6J mice showed higher *Giardia* colonisation with the faecal *Giardia* cyst number peaking at 6–7 dpi with a second peak at 10–11 dpi before cyst numbers stabilised (Figure 1B). Based on body weights, *G. lamblia* cyst count, and response to infection, C57BL/6J was selected for the follow-up main study with a shortened infection period of 10 days.

### 2.2. Gut Microbial Distribution during Giardiasis

Across the gut, ca. 1336 different sequences were identified using QIIME pipeline (min length—284 bp, Max length—319 bp, and average length—286 bp). The data represented 18 bacterial orders with 33 families. The primary analysis of the 16S rRNA sequences from all the samples showed the presence of the bacterial species from the *Muribaculaceae*, *Lachnospiraceae*, *Lactobacillaceae*, *Erysipelotrichaceae*, and *Ruminococcaceae* families. A detailed genus representation for the major bacterial families represented is shown in Figure S1. Of 18 orders, *Clostridiales* showed the highest representation, especially from *Lachnospiraceae* (568 genera) followed by *Runimococcaceae* (285 genera) families (Figure S1).

The change in microbial diversity within each part of the mice gut was detected using the Faith-PD index (Figure S2) which suggested that the microbial diversity was particularly affected in the ileum, followed by jejunum, during giardiasis. The dissimilarity between samples during all infections, measured using the Jaccard Emperor and Bray–Curtis dissimilarity indexes (Figure S3) showed two distinct clusters representing the small (duodenum, jejunum, and ileum) and large (caecum and colon) intestines.

**Figure 2:**
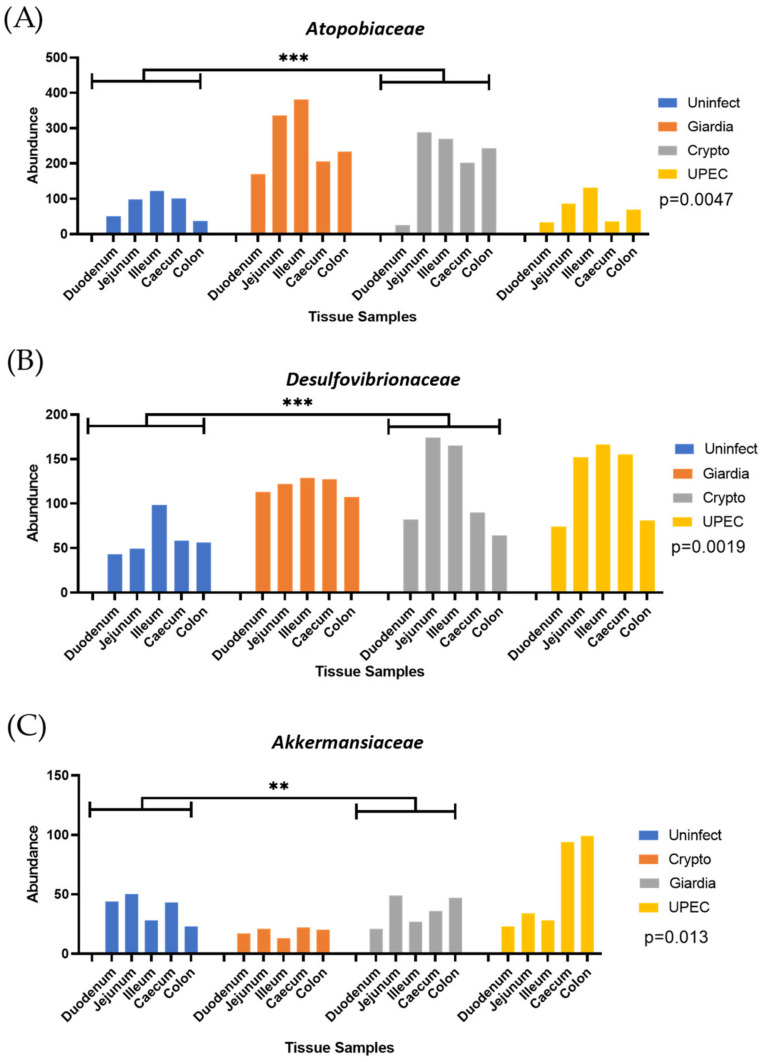
Relative population abundance of (**A**) *Atopobiaceae,* (**B**) *Desulfovibrionaceae*, and (**C**) *Akkermansiaceae* bacterial species which showed the most significant changes amongst all genera observed during giardiasis with respect to other infections and uninfected condition, with significance of *p* ≤ 0.01 (***) and *p* ≤ 0.05 (**).

PICRUST and BURRITO analysis [22,23] indicated that three microbial families (*Autopobiaceae*, *Desulfovibrionaceae*, and *Akkermanasiaceae*) exhibited significant population change during giardiasis. The *Atopobiaceae* (combined *p*-value = 0.005, Figure 2A) and *Desulphovibrionaceae* (combined *p*-value = 0.002, Figure 2B) families showed a significant increase in abundance throughout the gut during giardiasis. On the other hand, the abundance of *Akkermansiaceae* significantly decreased in the gut during giardiasis (combined *p*-value = 0.013, Figure 2C).

The predictive functional content analysis of the metagenome samples showed that significant change in microbial load across the gut system may have contributed towards altered metabolic pathways. The increased abundance of *Autopobiaceae* and *Desulphovibrionaceae* families during giardiasis was positively correlated with the expression of metabolic pathways involved in carbohydrate, lipid, amino acid, and energy-related pathways, particularly in the small intestine (Figures S4–S9 and S12–S15). The reduction in *Akkermansiaceae* abundance, particularly during giardiasis, predicted a likely negative impact on carbohydrate, lipid, energy, and amino acid metabolism (Figures S16 and S17 and Tables S2 and S3). The microbial distribution of key species affected in the gut during giardiasis is a potential discrimination tool to distinguish this infection from other bacterial (UPEC) and eukaryotic (cryptosporidiosis) parasites.

### 2.3. Gut Metabolism during Giardiasis

Metabolomics output showed the presence of 187 statistically significant metabolites across all the tested samples during all the infections. Proteomics analysis identified 2129 host proteins being expressed in the gut and 2038 host proteins expressed in serum and liver during giardiasis. The biomarker meta-analysis indicated that, of these, only 658 host proteins and 80 metabolites were expressed (upregulation or downregulation) with statistically significant difference across all the organs during giardiasis. Across all the samples and infections, microbial protein expression gradually increased in the luminal content as it passed through the intestine, with the ileum having the most microbial proteins (142 proteins) expressed. In the large intestine, 874 proteins were expressed in caecum, 815 in the colon, and 956 in the faeces, respectively. The integrated proteome–metabolome analysis indicated that the metabolism of carbohydrates, amino acids, lipids, and cofactors/vitamins were more prominent in the mouse gut during giardiasis with respect to other conditions studied. The integrated outputs of host proteome and metabolome indicated 12 major metabolic pathways across the gut (upregulated or downregulated) which were statistically significant (Table 1 and Table S1) during giardiasis with respect to other infections.

#### 2.3.1. Energy Pathways

**Figure 3:**
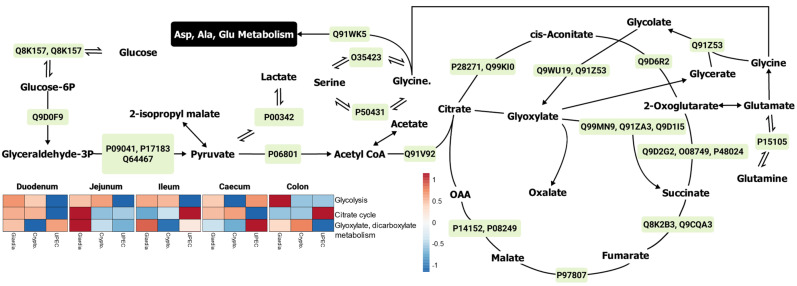
The major proteins (green boxes) and metabolites contributing to the energy metabolism in mouse gut during giardiasis. The heatmap represents Log_2_ Fold change-based relative upregulation (red) and downregulation (blue) of individual pathways in the mice with infected gut with respect to the uninfected mice. Note: The protein identifiers (green boxes) refer to the Uniprot IDs. The heatmaps were designed using the Clustvis web tool [24]. Details for major proteins (Figure S4) and metabolites (Table S2) are provided in the Supplementary Materials.

Overall, we observed that energy pathways, such as glycolysis and citrate cycle, were upregulated more in the small intestine during giardiasis compared with the large intestine. It was also apparent that glyoxylate and glycolate metabolism played an important role in diverting the metabolism of amino acids such as glutamate, glycine, and serine towards energy pathways in the small intestine, especially when comparing *Giardia* and UPEC infection (Figure 3). Protein expression indicated that the major glycolytic enzymes (Figure S4), such as phosphoglycerate kinase and isocitrate dehydrogenase (Figure S4A,H), showed similar upregulation during both giardiasis and cryptosporidiosis. On the other hand, galactose mutarotase, aminomethyl transferase, and methylmalonyl-CoA epimerase expression were similar during giardiasis and UPEC infection (Figure S4F–K).

#### 2.3.2. Pentose Phosphate Pathways (PPP) and Amino Acid–Sugar Interconversions

**Figure 4:**
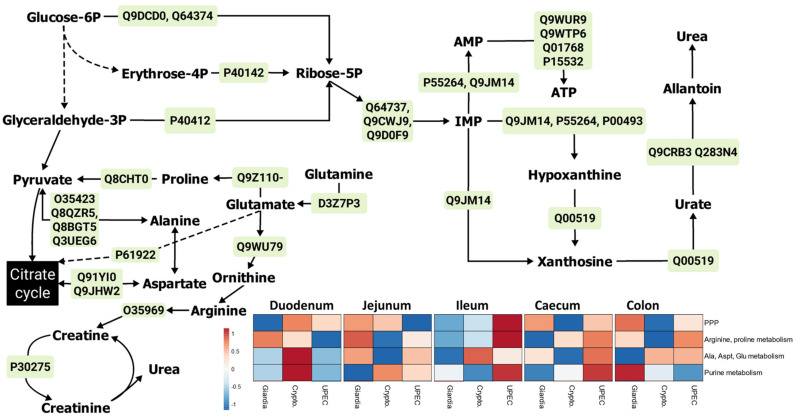
The major proteins (green boxes) and metabolites contributing to the pentose phosphate pathway and amino acid–sugar interconversions in mouse gut during giardiasis. The heatmap represents relative upregulation (red) and downregulation (blue) of individual pathways in the mice with infected gut with respect to the uninfected mice. Note: The protein identifiers (green boxes) refer to the Uniprot IDs. The heatmaps were designed using the Clustvis web tool [24]. Details for major proteins (Figure S5) and metabolites (Table S2) are provided in the Supplementary Materials.

The downstream pathways to energy pathways, such as PPP, appeared to be upregulated across the jejunum, caecum, colon, and faeces during giardiasis. The PPP appeared to be directly impacting the downstream phenylalanine pathway in the caecum and colon, and purine metabolism in colon (Figure 4 and Figure S5). On the other hand, non-aromatic amino acid metabolism showed relative downregulation throughout the different regions of the gut (Figure 4 and Figure S5), with the exception of jejunum during giardiasis (Figure 4). Although the major protein expression profile during giardiasis was similar to that of UPEC infection, proline dehydrogenase (Figure S7E) and creatine kinase (Figure S7G) were upregulated during giardiasis throughout the different gut regions.

#### 2.3.3. Redox (Sulphur-Related) Metabolism and Pathways

**Figure 5:**
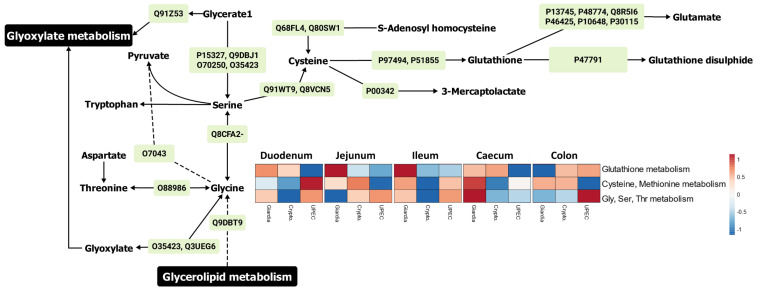
The major proteins (green boxes) and metabolites contributing to the sulphur-related redox metabolism in mouse gut during giardiasis. The heatmap represents relative upregulation (red) and downregulation (blue) of individual pathways in the mice with infected gut with respect to the uninfected mice. Note: The protein identifiers (green boxes) refer to the Uniprot IDs. The heatmaps were designed using the Clustvis web tool [24]. Details for major proteins (Figure S6) and metabolites (Table S2) are provided in the Supplementary Materials.

Glutathione metabolism was upregulated throughout the gut, with the exception of the colon, by the activity of both host (Figure 5 and Figure S6) and microbial proteins. The proteome-metabolome integration indicated that among the major proteins driving the glutathione metabolism, glutathione reductase (GLR1, P47791) showed upregulation in the large intestine (Figure S6A). Although the glutathione S-transferases were more active in the large intestine during giardiasis, some proteins of this class, such as glutathione S-transferases were upregulated in the small intestine as well (Figure S6D,E,G). Furthermore, glutathione synthetase expression was downregulated during giardiasis, with respect to the other infections (Figure S6H), especially in the jejunum, ileum, and colon. Another important upregulated enzyme was membrane primary amine oxidase (Figure S6L) which catalyses glycine-to-pyruvate conversions, indicating the diversion of amino acids into energy pathways through glycolysis.

#### 2.3.4. Short-Chained Fatty Acid (SCFA) and D-Amino Acid Metabolism

**Figure 6:**
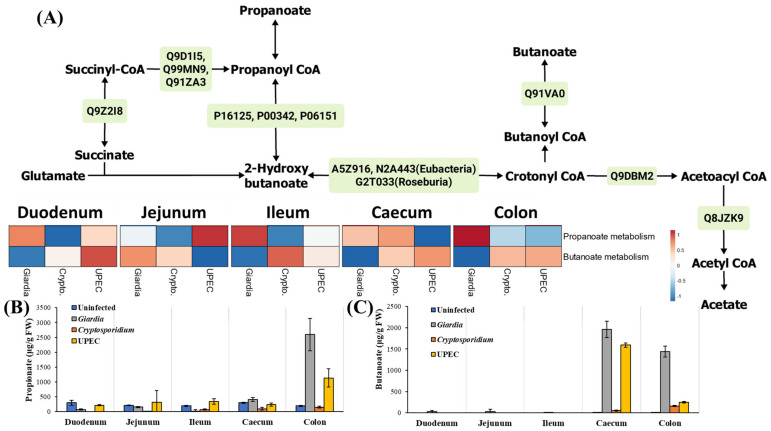
The (**A**) major proteins (green boxes) and metabolites contributing to fatty acid metabolism in mouse gut during giardiasis. The heatmap represents relative upregulation (red) and downregulation (blue) of individual pathways in the mice with infected gut with respect to the uninfected mice. Note: The protein identifiers refer to the Uniprot IDs. (**B**) Propanoate and (**C**) butanoate expression in gut.

The major fatty acid pathways expressed in the gut during giardiasis were propionate and butanoate metabolisms. Particularly, propanoate metabolism was observed to be upregulated in the gut (except in jejunum) during giardiasis with respect to other infections (Figure 6A). Propanoate and butanoate levels were also increased in the large intestine and faeces during giardiasis (Figure 6B,C). Furthermore, D-amino acid accumulation was also observed to be higher in the small intestine, with a gradual decrease from the caecum onwards. However, when compared between the infected groups, considerably higher levels of D-amino acids such as D-alanine and D-proline were accumulated in the large intestine during giardiasis (Figure S8).

### 2.4. Extra-Gut Effects of Giardiasis

The integrated proteome–metabolome analysis of serum and liver indicated that 16 pathways were altered in a statistically significant manner due to gut infections (Table S2). It was observed that in serum, energy pathways such as glycolysis and citrate cycle, and immediate supporting pathways, such as glutathione metabolism and PPP, were upregulated during giardiasis with respect to cryptosporidiosis and UPEC infection (Figures S9 and S10). While the glutathione pathway was upregulated throughout the gut and serum, this trend was not observed in the liver. On the other hand, the upregulation of PPP in the serum showed an inverse correlation to that in the gut environment. Arginine and proline metabolism also showed greater upregulation in the serum and liver, with respect to the gut (Figures S9A,B, Tables S3–S5).

**Figure 7:**
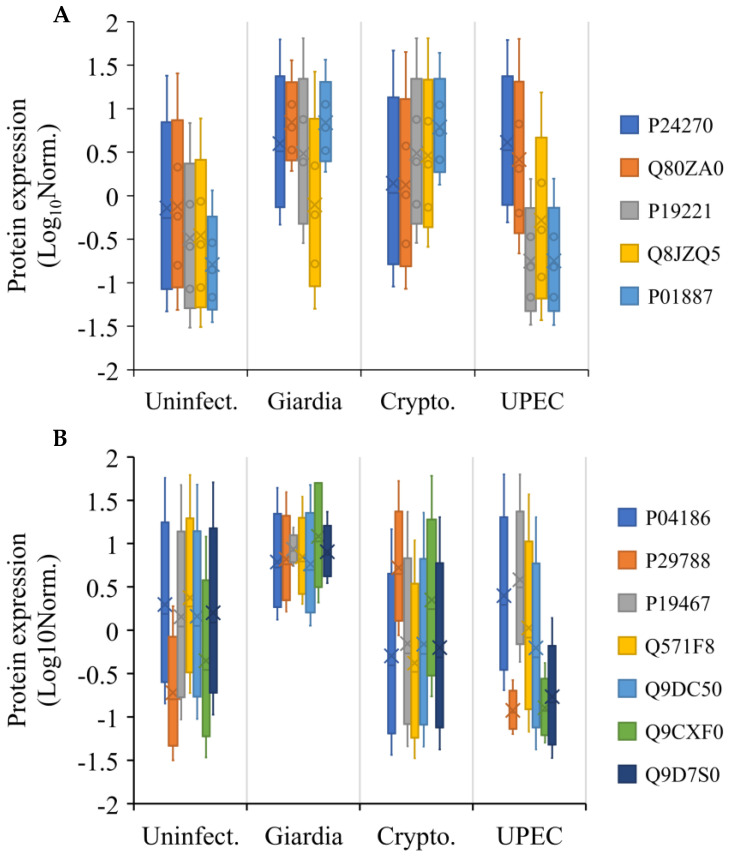
Log_10_ normalised expression of proteins from (**A**) serum and (**B**) liver which were observed to be involved in cellular protection as an extra-intestinal effect of various gut infections in the mouse model. Note: The protein identifiers refer to the Uniprot IDs.

We observed that the proteins related to stress response and anti-inflammatory activities were upregulated in both serum and liver. In serum, these proteins included intelectin-1b (Q80ZA0), catalase (P24270), and β-2-microglobulin (P01887), which showed greater expression during giardiasis with respect to other infections (Figure 7A). In the liver, proteins upregulated during giardiasis included vitronectin (P29788), Ly6/PLAUR domain protein (Q9D7S0), complement factor B (P04186), and mucin-13 (P19467) (Figure 7B).

## 3. Discussion

A recent post-transcriptional regulation study [25] has shown *Giardia* to be one of the earliest evolutionary lineages among eukaryotes, even with respect to *Saccharomyces cerevisiae*, the simplest known eukaryotic model. As such, some of the key metabolic pathways during giardiasis may have a similar expression level to infection by more evolved protists such as *Cryptosporidium*, or to either yeast or bacteria. In this study, we showed that giardiasis results in greater expression of energy and associated pathways in the duodenum–jejunum and colon. This was somewhat different from the mechanism during cryptosporidiosis, where these pathways were greatly upregulated in the jejunum–ileum [26,27]. It has been shown that the microaerophilic nature of *Giardia* aids in modulating the energy pathways during increased oxygen tension [12]. The upregulated energy pathways extending to non-gut regions, such as serum and liver, were expected as the liver (part of the gut–liver axis) is known to reflect the pathologic effects of gut infections and disorders [28]. The upregulated glutaminase activity not only caused the energy pathway upregulation, but also suggested increased ammonia levels [29] and triggered the upregulation of amino acids through the arginine and proline metabolism pathways [30,31]. Although these results indicate a possible liver injury, histological experiments would be able to confirm these observations.

The 16S metagenome data provided insight into possible metabolic changes taking place during giardiasis and cryptosporidiosis, highlighting the potential for these tools to explain dynamic tissue metabolism. The small intestine is more active in digestion and nutrient absorption processes compared with the large intestine [32], as shown in this study by the PICRUST analysis where all the carbohydrate, lipid, amino acid, and energy-related pathway activities were increased only within duodenum, jejunum, and ileum. The predicted activity change was limited in the large intestine (cecum and colon). During giardiasis, an increased abundance of Firmicute anaerobes such as *Lactobacillus*, *Ruminococci,* and *Desulphovibrio* is generally reported in the small intestine [18], very likely due to the increased redox perturbations. Our integrated approach confirmed these predictive observations through the functional omics, as shown through the increased abundance of *Atopobiaceae*, *Desulfovibrionaceae,* and decreased abundance of *Akkermansiaceae* during giardiasis. These perturbations are primarily caused by the altered glutathione (GSH)/glutathione disulphide (GSSG) ‘oxidation ⇆ reduction (redox)’ potential. Our results showed significantly high expression of glutathione, cysteine, and methionine metabolism during giardiasis and are consistent with the recent murine [33] and cell culture [34] studies which showed elevated GSH expression.

Million et al. [35] showed that an increased redox potential created by elevated glutathione causes the production of SCFAs such as butanoate in the gut epithelium. The thiol-cycling enzymes of *Giardia* and genes related to oxidoreductase metabolism have been shown to progressively downregulate under axenic conditions [36]. This results in increased expression of sulphur-related metabolism as a gut modulation, causing *Giardia*-induced injuries and upregulation in apoptosis-related proteins. In the current study, upregulation of both apoptosis-related proteins and cell-protective proteins (Figure S11 correlated with the upregulation of glutathione metabolism enzymes (Figure S6). A direct correlation between the upregulated catalase activity and glutathione metabolism with respect to the increased immune response during lipopolysaccharide (LPS)-induced oxidative damage and caspase-dependent apoptosis has been demonstrated in the fish gut [37] and in the intestinal epithelial cell culture experiments [38]. The elevated glutathione and redox pathways could also be attributed to the increase in the *Desulfovibrio* populations. It has been shown that sulphate is utilised by sulphate-reducing bacteria (SRB) such as *Desulfovibrio* as a terminal acceptor for fatty acids, lactate, or hydrogen oxidation [39]. This alteration in gut metabolism by SRBs is known to trigger inflammation through epithelial apoptosis [40], leading to disorders such as ulcerative colitis [41]. Our results also indicated that *Desulfovibrio* populations likely caused a depletion of *Akkermansiaceae* population. *Akkermansia* populations are important species that provide anti-inflammatory effects in the gut and beyond. The bacteria are also known to cause downregulation of pro-inflammatory cytokines such as TNF-α and IFN-γ in mouse gut [16,42].

The oxidative stress observed in the gut was found to extend to non-gut environments. Upregulated glutathione metabolism (Figure S6, Table S2) was demonstrated by the observation of depleted glutathione and upregulated glutathione and cysteine–methionine metabolising enzymes (Figure S6) in the serum in the current study. The observations were in line with previously reported blood biochemistry, especially the significant depletion of glutathione and upregulated catalase in the blood samples of patients infected with *Helicobacter pylori* [43]. This mechanism is exploited in anti-*Giardia* drugs, such as NBDHEX, for inhibiting *Giardia* disulphide reductase and elongation factor proteins [34].

Similar to the glutathione metabolism, nitric oxide (NO) accumulation is also seen as an effect of the oxidative stress induced by gut infection [44]. Recent studies of chicken ileum have indicated an increase in NO during inflammation and oxidative stress [45]. It has been shown that NO is produced as a by-product of arginine catabolism and the consumption of arginine by *Giardia* leads to the inhibition of NO production [46,47], causing downregulated arginine metabolism and interference with the host immune response. The current study suggests that the non-depleted arginine and proline metabolism was due to the metabolic alterations caused by ammonia accumulation and may lead to liver injury [29,31] during giardiasis. The possibility of liver injury and inflammation is supported by the upregulated intelectin −1 (ITLN-1) [48,49] and Ly6/PLAUR Ly6/PLAUR activities in serum and liver, respectively. Expression of Lys6/LAUR and mucin protein during ulcerative colitis [50] and apicomplexan infection [51] have been observed in the colonic mucosa. However, the current study indicates that their effects extend beyond the intestine to the entire gut–liver axis. In addition to the host response to infection, bile acid metabolism and mitochondrial oxidation in the gut–liver axis have been shown to be upregulated by the activities of *Akkermansiaceae* family bacteria. Indeed, supplementation of *Akkermansia muciniphila* has been shown to improve gut–liver interactions and ameliorate hepatic injury [52] by elevating L-aspartate transport from gut to liver.

It has previously been shown that parasitic infection disrupts the normal gut microbiome [53]. SCFAs [16,17] and D-amino acids, which are known to be produced by the gut microbiota, are known to play an important role in gut immunomodulation [54]. Recent studies have shown that SCFAs such as propionate are transported from the gut to the liver via portal circulation. Metabolism of SCFAs by hepatocytes [55] has shown to cause elevated gluconeogenesis and downregulated pro-inflammatory proteins such as interleukin-8 [56], pyruvate recycling accompanied by elevated anaplerosis, and mitochondrial metabolism [57]. Recently, mRNA expression analysis [58] demonstrated the impacts of butyrate in upregulating the serum IL-10 and IgM activities, and arginine and tryptophan metabolism. The predicted minor increase in lipid metabolism also supports previous findings of the positive correlation of *Coriobacteriaceae* abundance with cholesterol and triglyceride concentration [59]. The role of these bacteria in anti-inflammatory activity has been suggested recently [60]. The increased predicted metabolic activity in amino acid metabolism by *Atopobiaceae* in the current study supports previous findings of increased lactate production and increased aminopeptidase activity by *Coriobacteriaceae* (*Atopobiaceae*) [61].

## 4. Materials and Methods

### 4.1. Animal Ethics Approval

Animal handling and experimentation was performed in accordance with Victorian State Government regulations and Animal Research: Reporting of In Vivo Experiments (ARRIVE) [62] and was approved by the Monash University Animal Ethics Committee (Monash University AEC no. MARP/2018/055 and 15236).

### 4.2. Mouse Infection and Strain Selection

*Giardia lamblia* was obtained from BTF bio (Cat. Number: G10E6-V; BTF Pty Ltd., North Ryde, NSW, Australia). Murine susceptibility to *G. lamblia* was assessed using C57BL/6J, BALB/c, and Swiss mice (n = 3). During 7-day acclimation and study periods, the mice were housed in Optimice cages containing sterile sawdust (18–24 °C, 40–70% humidity, 12:12 h light/dark cycle). Feed and water were available ad libitum. Mouse feed was obtained from Ridley Agriproducts (Barastoc WEHI mouse breeder cubes, Product code: 102119, Ridley AgriProducts Pty. Ltd., Melbourne, VIC, Australia). Female mice (age = 3 weeks) were infected with 1 × 10^5^ oocysts via oral gavage [27] and monitored for 14 days. Faecal samples were collected daily. Blood was collected by cheek bleed at 0- and 7-days post-infection (dpi) and by cardiac bleed after euthanasia (CO_2_ exposure) at 14 dpi, with tissues collected thereafter. Serum and liver were used as representatives for the indirect and cross-organ effects of *G. lamblia* infection. Luminal washes of duodenum, jejunum, ileum, caecum, and colon were collected by flushing with 1 mL sterile phosphate buffer saline and used as the intestinal representatives. The samples were immediately stored on dry ice to arrest metabolism and then at −80 °C until further analysis. The infection was quantified by the detection of oocysts and *G. lamblia* genomic material by fluorescent microscopy using the EasyStain kit (BTF Pty Ltd., North Ryde, NSW, Australia).

### 4.3. Microbiome, Proteomics, and Metabolome: Extraction and Processing

Microbial, protein, and metabolic extractions from luminal washes, blood serum, and liver were performed as per the protocols reported previously [27] without any modifications. Amplicons were generated from the V3 and V4 regions of 16S rRNA using gene-specific primers. The purified library was sequenced and demultiplexed on an Illumina MiSeq using a v3 300 bp PE sequencing kit following the manufacturer’s protocol. Sequences processing was performed with the QIIME 2 (Release no. 2019.7) pipeline [63] similar to previously reported methodology [18] using the Silva138 database [64]. Amplicon sequence variants (ASVs) data were clustered at 97% sequence similarity, using USEARCH -cluster_fast on size (abundance) sorted ASVs, for prediction of the metagenomic function with PICRUSt2 [22] using the previously reported method [19].

The protein concentration was determined by Bradford assay. Following this, 5 µg of protein was taken for further processing. Volume was adjusted to 10 µL by adding urea (8 M). Dithiothreitol (1 µL, 15% *v*/*v*) was added, followed by a 30 min room temperature incubation. Bis-acrylamide (1 µL, 40% *v*/*v*) was then added and followed with a further 30 min incubation at room temperature. Trypsin (0.1 µg in 20 mM ammonium bicarbonate) was added, and the mixture incubated for 3 h at 37 °C. Formic acid (1 µL, 10% *v/v*) was added to terminate trypsin activity. Tryptic peptides (100 ng) were desalted and concentrated with a trap column (PepMap100 C18 5 mm × 300 µm, 5 µm) and separated on a nano column (PepMap100 C18 150 mm × 75 µm, 2 µm) using an Ultimate^TM^ 3000 RSLC nano LC system, with mobile phases (A: water + 0.1% (*v*/*v*) formic acid; B: acetonitrile (80% *v*/*v*) + 0.08% (*v*/*v*) formic acid). The peptides were eluted using Solvent B at gradients of 5–40% (0–60 min) and 40–99% (60–70 min). The eluted peptides were ionized with a Nanospray Flex Ion Source. Proteins were analysed and normalised as per the methods described previously [27] without any modification against the Uniprot *Mus musculus* protein database (UP000000589).

For metabolomic analysis, the samples were subjected to in-time derivatisation on an Agilent 7890 B gas chromatography system with a 5977 B mass spectrometry detector fitted with an MPS autosampler (Gerstel GmbH & Co. KG, Mülheim an der Ruhr, Germany) as per the previously reported configurations and settings [14].

For microbiome analysis, mouse faeces and luminal contents (n = 5 each) were homogenised, and DNA was extracted using the manufacturer’s instructions (ZymoBiomics DNA miniprep kit, Zymo Research Corp., Irvine, CA, USA). The 16S rRNA amplification and Illumina sequencing followed by the analysis on the QIIME 2.0 platform were performed using the protocols reported previously [27,60] using the SILVA database V.138.

### 4.4. Statistical Analysis and Quality Control

The raw data for metabolites obtained from the MassHunter workstation were subjected to the batch effect adjustment tool of MetaboAnalyst 5.0 [65]. The batch effect adjusted data were further normalised to the IS2 (Myristic acid-d_27_, 10 µg per sample, relative standard deviation (RSD) = 8.26%). Similarly, variability between the samples was indicated by the RSD of IS1 (valine-^13^C_2_ = 7.86% and stearic acid-^13^C = 1.87%). Additionally, the metabolic output was further normalised according to the sample weights and was expressed as metabolite concentration (µg/g fresh sample weight). Normalisation for proteomics dataset was performed as per the method previously described [27].

The normalised proteomic and metabolomic data were analysed using multivariate data analysis software SIMCA (version 14.1, Sartorius Stedim Biotech, Umeå, Sweden). The R^2^X and R^2^Y values defined the variation between X and Y variables of various components in the sample set and Q^2^ gives predictability of the model [66]. Furthermore, univariate statistical tools such as volcano plots, enrichment analysis, and pathway impacts were measured by MetaboAnalyst 5.0 [65]. The cut-off level for significant metabolites was a signal-to-noise (S/N) ratio of 10, a fold change of ≤0.5 (downregulation) or ≥2.0 (upregulation), and a Benjamini–Hochberg adjusted *p*-value of ≤0.05.

### 4.5. Multi-Omics Integration

The taxonomy-to-phenotype mapping of gut microbiome and metabolome was conducted using Burrito [23] and MIMOSA2 [67], web-based systems biology tools, using the previously reported method [19]. The overall diversity representation was analysed by faith PD index for each sample. Due to the non-replicate structure of the experiments, the two-factor ANOVA without replication method was used to determine significant differences between treatment groups across gut samples (duodenum, jejunum, ileum, caecum, colon luminal washes, and faecal pellet). After diversity analysis, the predictive functional implications of metagenomes present in each tissue sample were analysed using the PICRUST program and Shiny-Burrito online platform. The sequencing efficiency was determined by comparing the percentage of different OTUs identified in the microbial community standard II sample (log distribution) (ZymoBiomics D6310, Zymoresearch Corp., Irvine, CA, USA)

The proteomic–metabolomic integration was performed in a two-step process. The datasets were combined through the ‘Biomarker meta-analysis’ tool of Metaboanalyst 5.0. A minimum threshold of *p*-value (*p* ≤ 0.05) and combined log fold change (combinedLogFC ≥ 1.0) were defined for both categories. The data obtained were subjected to proteomic–metabolomic integration and networking through the ‘Joint pathway analysis’ tool (Metaboanalyst 5.0) and Paintomics 3.0 web toolbox. The metabolic pathway mapping was performed by using the Omix visualisation tool (Version 1.9.34; Omix Visualisation GmbH and Co. KG, Lennestadt, Germany).

## 5. Conclusions

We studied the effects of giardiasis with respect to cryptosporidiosis and UPEC infection in a mouse model utilising integrated 16S rRNA genetics–metabolomics and proteomics–metabolomics multiomics platforms. Univariate and multivariate analyses indicated key pathways that were altered along the gut–liver axis during giardiasis. It appeared that both microbial perturbation and host responses contributed to upregulated energy pathways and fatty acid metabolism. Additionally, the oxidative stress triggered the upregulation of glutathione metabolism in the small intestine and liver, suggesting an activation of the redox pathway as a stress response mechanism. Our observations highlight the capability of multiomics integration to ascertain a comprehensive understanding of host–parasite interaction throughout the gut, and the previously unreported effects of these interactions on the gut–liver axis. The output of this study can be used to assess the specific unreported biochemical assays that can help to develop rapid, non-invasive biomarker panels and development of more effective treatment solutions for giardiasis. The outputs of this study will also potentially aid towards development of precision medicine for gut infections.

## Figures and Tables

**Table 1:** Major statistically significant pathways contributing towards the mouse gut metabolism during giardiasis, as determined by the joint pathway analysis.

Pathway	Duodenum		Jejunum		Ileum		Caecum		Colon		Faeces	
	Impact	FDR	Impact	FDR	Impact	FDR	Impact	FDR	Impact	FDR	Impact	FDR
Aminoacyl-tRNA biosynthesis	0.38	6.3 × 10^−6^	0.41	6.62 × 10^−6^	0.41	8.37 × 10^−6^	0.41	2.06 × 10^−5^	0.44	9.07 × 10^−6^	0.33	0.0001
Glyoxylate, dicarboxylate metabolism	1.05	4.3 × 10^−5^	1.16	5.64 × 10^−7^	0.91	7 × 10^−5^	1.16	1.33 × 10^−6^	1.35	4.18 × 10^−9^	1.13	4.85 × 10^−5^
Alanine, aspartate, and glutamate metabolism	1.17	0.0038	1.10	0.0024	1.07	0.0010	1.20	2.06 × 10^−5^	1.15	0.0008	0.82	0.0012
Arginine biosynthesis	0.88	0.0039	1.12	1.11 × 10^−5^	1.12	0.0006	1.31	1.33 × 10^−6^	1.35	1.69 × 10^−5^	0.85	0.0010
Glutathione metabolism	0.95	4.26 × 10^−7^	0.84	3.43 × 10^−6^	1.00	1.99 × 10^−6^	1.16	1.44 × 10^−5^	0.96	7.43 × 10^−6^	0.87	0.0001
Citrate cycle (TCA cycle)	1.63	0.0027	2.44	2.07 × 10^−7^	1.95	0.0007	2.07	0.0002	2.66	3.89 × 10^−6^	1.56	0.0022
Glycolysis or gluconeogenesis	1.17	0.0446	1.95	3.92 × 10^−13^	1.60	0.0006	1.55	0.0001	1.68	0.0004	1.48	0.0010
Pantothenate and CoA biosynthesis	0.73	0.0378	0.79	0.0398	0.79	0.0210	0.79	0.0084	0.73	0.0233	0.36	0.0878
Glycine, serine, and threonine metabolism	1.46	0.0275	1.63	0.0001	1.44	0.0027	1.66	0.0003	1.63	0.0028	1.48	0.0010
Phenylalanine metabolism	0.87	0.1507	0.96	0.0708	0.61	0.2560	1.22	0.0032	1.22	0.0099	0.78	0.0350
Pentose phosphate pathway	0.78	0.5734	1.48	0.0315	1.26	0.0021	1.22	0.0044	1.13	0.0197	1.13	0.0126
Butanoate metabolism	1.18	0.0027	0.93	0.0509	1.14	0.0027	1.11	0.0303	1.18	0.0016	0.25	0.2035

## Data Availability

Not applicable.

## Supplementary Materials

The supporting information can be downloaded at: https://www.mdpi.com/article/10.3390/ijms24021636/s1.
