# Supplementary material for: Gut Microbial Perturbation and Host Response Induce Redox Pathway Upregulation along the Gut–Liver Axis during Giardiasis in C57BL/6J Mouse Model
Source: Int J Mol Sci. 2023 Jan 13;24(2):1636. doi: 10.3390/ijms24021636 (PMC9862352; doi:10.3390/ijms24021636)
Supplement: Supplementary file 1 [file ijms-24-01636-s001.zip › ijms-2112261-supplementary.pdf]

Title

Glycolysis and oxidative stress related redox pathway upregulation along the gut-liver axis by gut microbial perturbation and host response during giardiasis in C57BL/6J mouse model

Authors

Avinash V Karpe[1], Melanie L Hutton[2], Steven J Mileto[2], Meagan L James[2], Chris Evans[2], Amol B Ghodke[3,4], Rohan M Shah[1,5], Suzanne S Metcalfe[1], Jian-Wei Liu[6], Tom Walsh[6], Dena Lyras[2,†], Enzo A Palombo[5,†], David J Beale[1,†,*]

[1] Land and Water, Commonwealth Scientific and Industrial Research Organization, Ecosciences Precinct, Dutton Park, QLD 4102, Australia.

[2] Infection and Immunity Program, Monash Biomedicine Discovery Institute and Department of Microbiology, Monash University, Clayton, VIC 3168, Australia.

[3] Queensland Alliance for Agriculture and Food Innovation, Department of Horticulture, The University of Queensland, St Lucia, QLD 4072, Australia

[4] Health and Biosecurity , Commonwealth Scientific and Industrial Research Organization, Ecosciences Precinct, Dutton Park, QLD 4102, Australia.

[5] Department of Chemistry and Biotechnology, School of Science, Computing and Engineering Technologies, Swinburne University of Technology, Hawthorn, VIC 3122, Australia.

[6] Land and Water, Commonwealth Scientific and Industrial Research Organization, Agricultural and Environmental Sciences Precinct, Acton, Canberra, ACT 2601, Australia

Infection analysis and mouse strain selection

The mouse strain with optimal for infection and *Giardia* colonisation among the tested strains, was selected by conducting the pilot study. During the study period of 14 days, the mice did not show visual diarrheic effects. Also, visual inspections and weight measurements (Figure 1A) indicated that the health of the mice was not drastically affected. Swiss mice showed considerable weight gains, with an average increase of 3.02 g during study period. Body weights of C57BL/6J and BALB/C mice, although fluctuate up to 8 dpi, did not increase considerably, with an average increase on 0.45 g and 0.11 g, respectively. C57BL/6J however, showed the highest response to *Giardia* infection, with the faecal *Giardia* cyst number peaking at 6 – 7 dpi (Figure 1B).

On 10 – 11 dpi, the *G. lamblia* cyst count showed a second peak before stabilising. Based on the outputs of both mice weights, *G. lamblia* cyst count and response to the infection, C57BL/6J strain was selected, with the follow-up main study shortened to 10 dpi.

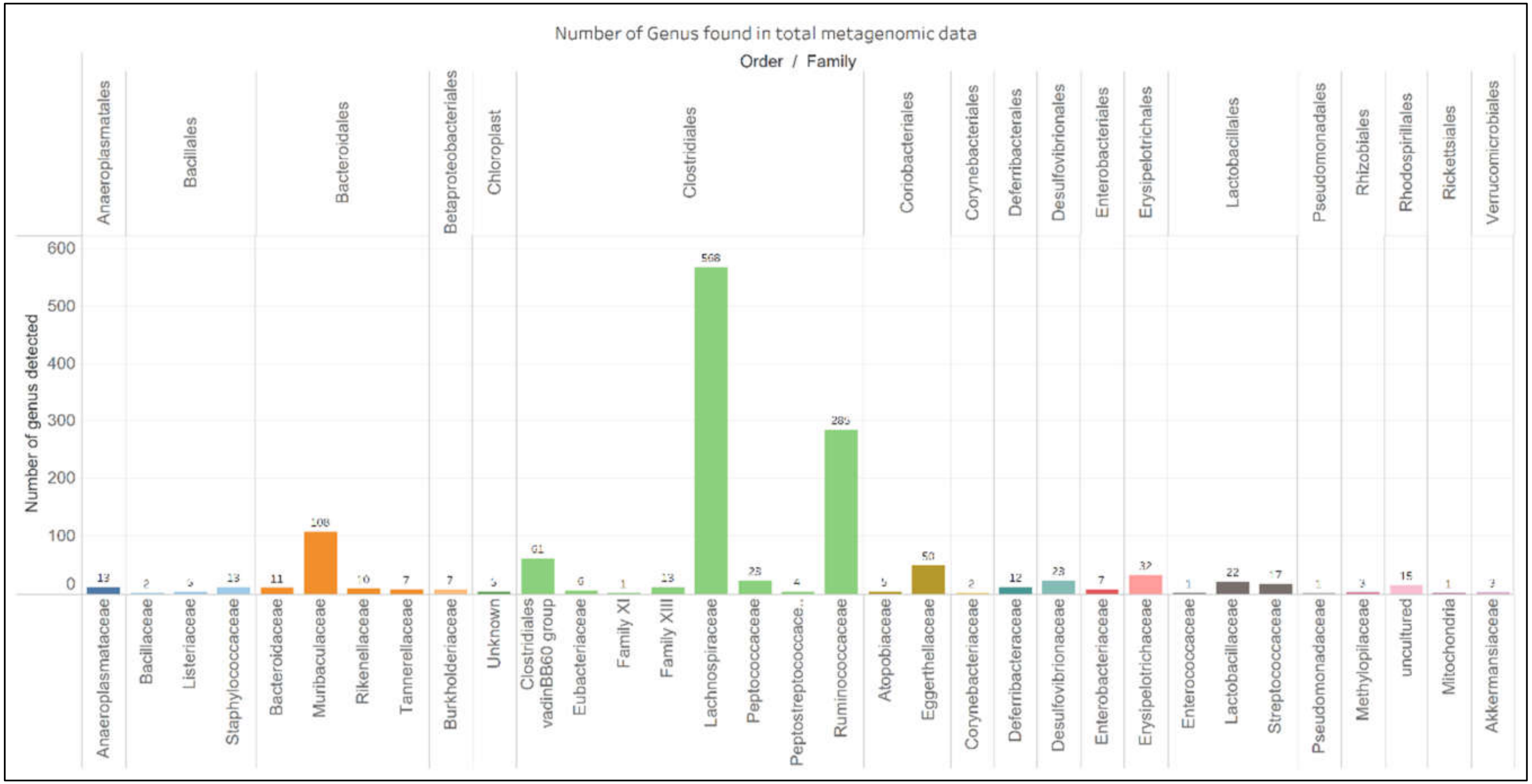

Figure S1. – Number of genera detected for each bacterial family. *Clostridiales* showed highest number of genera, followed by *Bacteroidales*. *Lachnospiraceae*, *Ruminococcaceae* and *Muribaculaceae,* in the mouse gut system during giardiasis

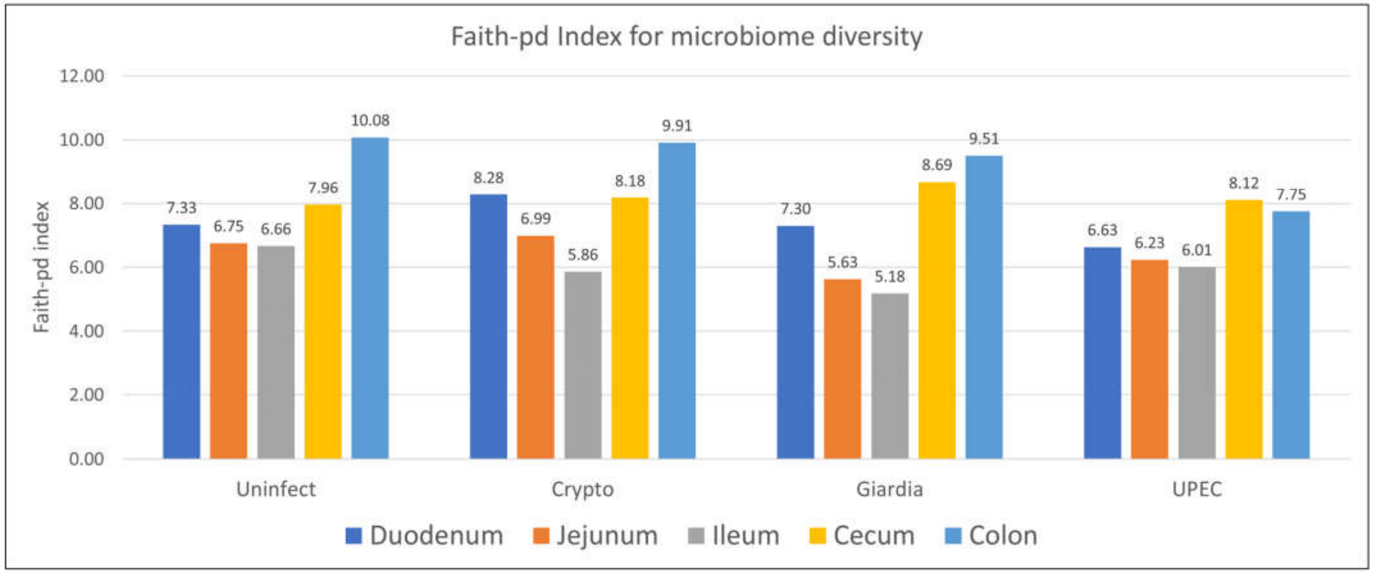

Figure S2. – Faith-pd index indicating overall microbial index detected in parts of the gut after specific diet treatment. Ileum was found most affected part of the gut showing decrease in the overall diversity during cryptosporidiosis and giardiasis.

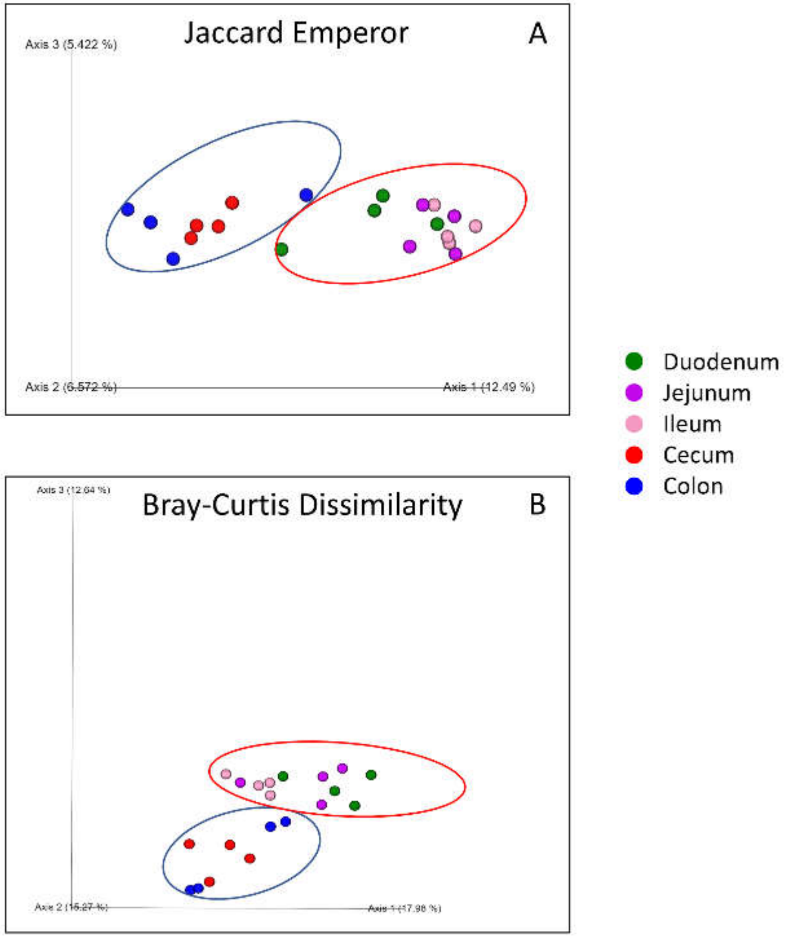

Figure S3. Jaccard Emperor (A) and Bray-Curtis Dissimilarity PCoA plots showing dissimilarity between small intestine (Duodenum, Jejunum and Ileum) and large intestine (Cecum and Colon). Axis 1 represents maximum dissimilarity followed by axis 2 and axis 3 between samples

Table S1. PLS-DA model fit (R^2) and predictability (Q^2) for metabolite profiles of all samples analysed for *Giardia* infection.

**Sample**	**R^2X**	**R^2Y**	**Q^2**
Faeces	0.802	0.997	0.985
Duodenum wash	0.682	0.999	0.954
Jejunum wash	0.65	0.994	0.865
Ileum wash	0.611	0.996	0.915
Caecum wash	0.702	0.999	0.975
Colon wash	0.603	0.996	0.972
Serum	0.691	0.996	0.980
Lung	0.659	0.989	0.929
Liver	0.574	0.999	0.955

Host protein expression during energy pathways (Glycolysis, Citrate cycle and Glyoxylate and gluconate pathway)

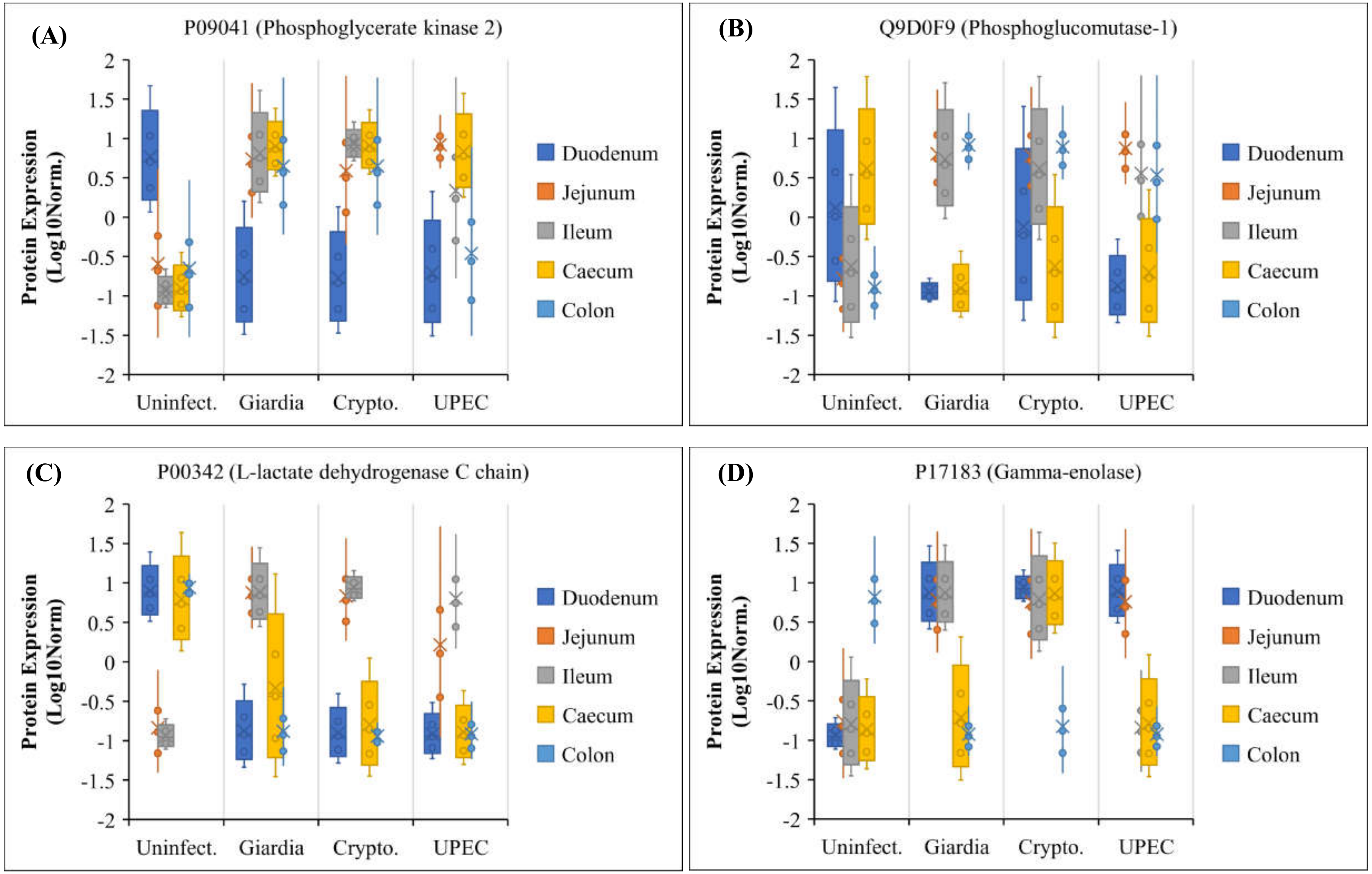

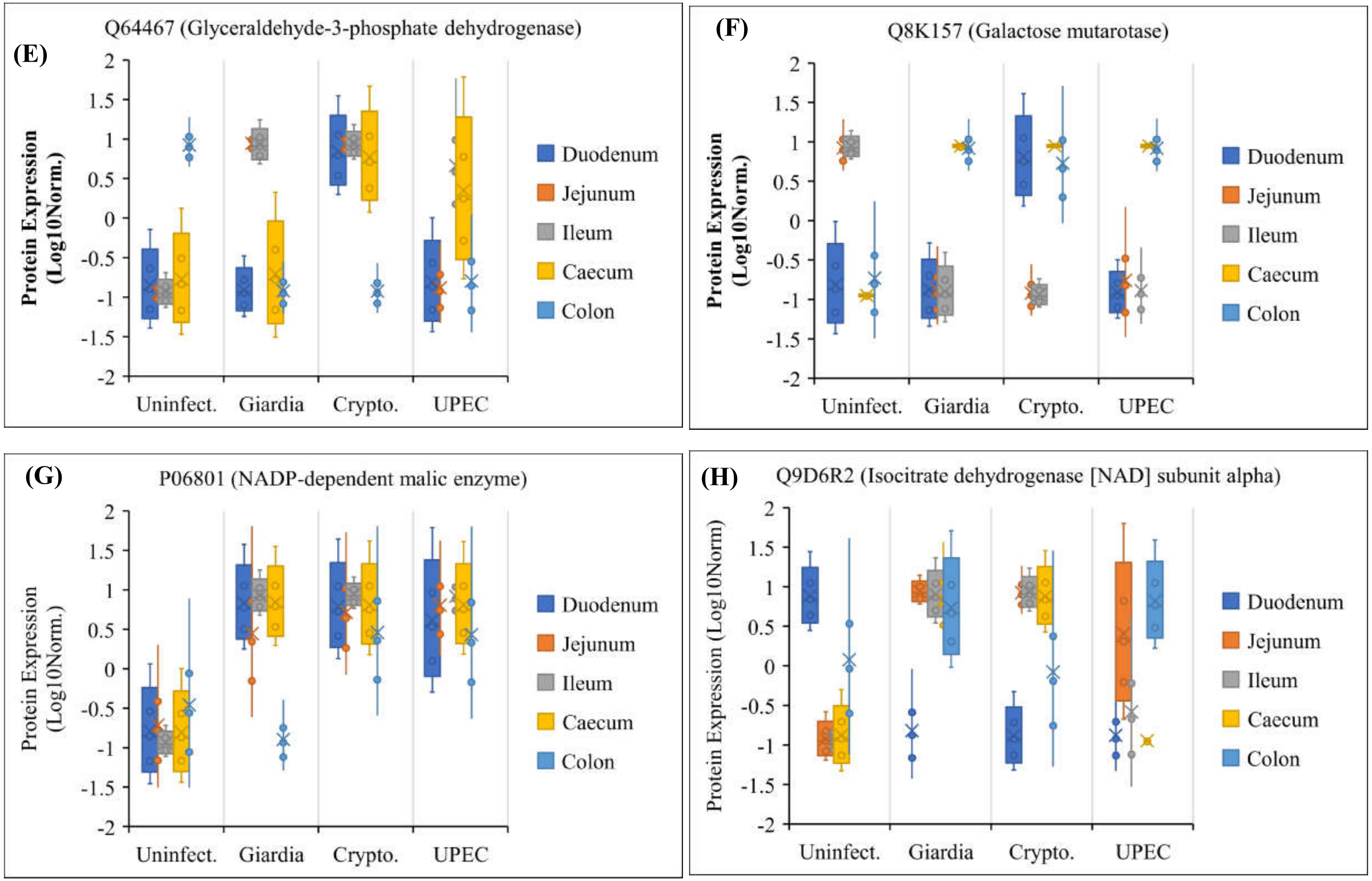
(E)
Q64467 (Glyceraldehyde-3-phosphate dehydrogenase)
Protein Expression (Log10Norm.)
2
1.5
1
0.5
0
-0.5
-1
-1.5
-2
Uninfect.
Giardia
Crypto.
UPEC
Duodenum
Jejunum
Ileum
Caecum
Colon
(F)
Q8K157 (Galactose mutarotase)
Protein Expression (Log10Norm.)
2
1.5
1
0.5
0
-0.5
-1
-1.5
-2
Uninfect.
Giardia
Crypto.
UPEC
Duodenum
Jejunum
Ileum
Caecum
Colon
(G)
P06801 (NADP-dependent malic enzyme)
Protein Expression (Log10Norm.)
2
1.5
1
0.5
0
-0.5
-1
-1.5
-2
Uninfect.
Giardia
Crypto.
UPEC
Duodenum
Jejunum
Ileum
Caecum
Colon
(H)
Q9D6R2 (Isocitrate dehydrogenase [NAD] subunit alpha)
Protein Expression (Log10Norm)
2
1.5
1
0.5
0
-0.5
-1
-1.5
-2
Uninfect.
Giardia
Crypto.
UPEC
Duodenum
Jejunum
Ileum
Caecum
Colon

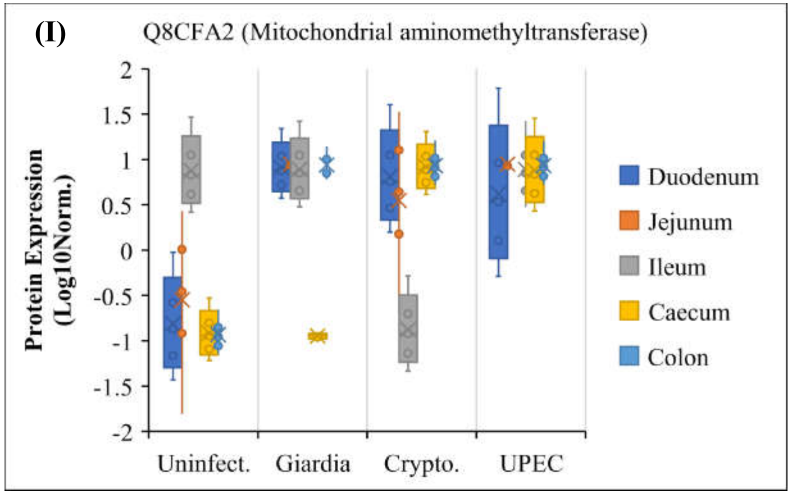
(I)
Q8CFA2 (Mitochondrial aminomethyltransferase)
Protein Expression (Log10Norm.)
2
1.5
1
0.5
0
-0.5
-1
-1.5
-2
Uninfect.
Giardia
Crypto.
UPEC
Duodenum
Jejunum
Ileum
Caecum
Colon

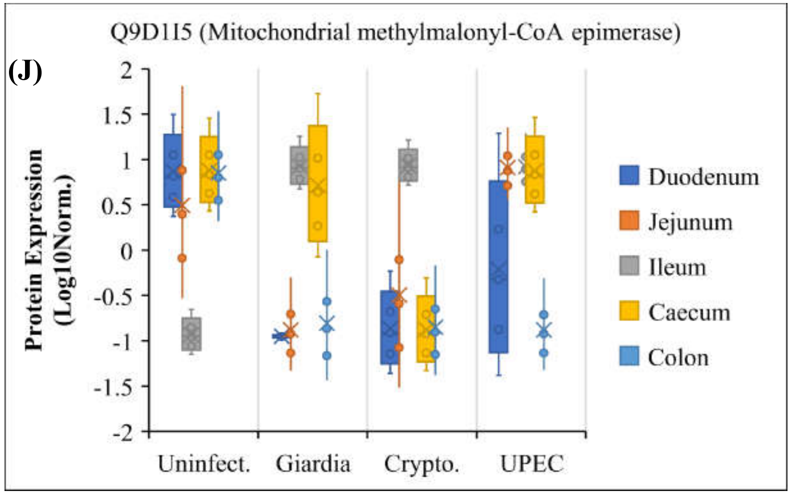
Q9D1I5 (Mitochondrial methylmalonyl-CoA epimerase)
(J)
Protein Expression (Log10Norm.)
2
1.5
1
0.5
0
-0.5
-1
-1.5
-2
Uninfect.
Giardia
Crypto.
UPEC
Duodenum
Jejunum
Ileum
Caecum
Colon

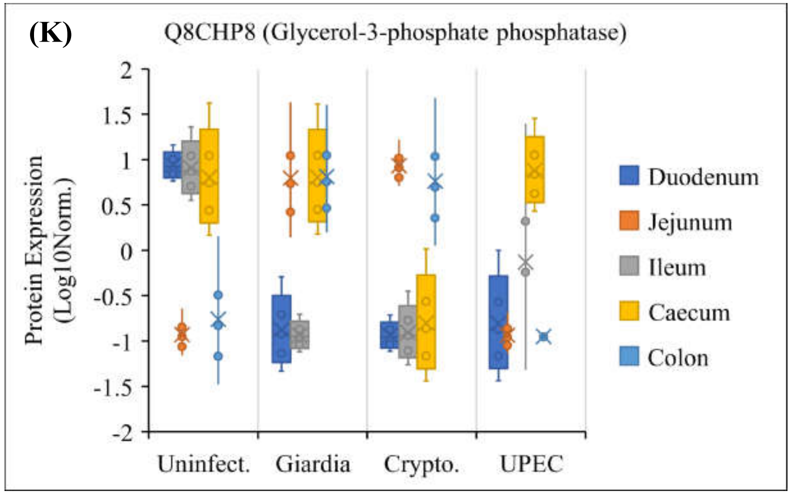
(K)
Q8CHP8 (Glycerol-3-phosphate phosphatase)
Protein Expression (Log10Norm.)
2
1.5
1
0.5
0
-0.5
-1
-1.5
-2
Uninfect.
Giardia
Crypto.
UPEC
Duodenum
Jejunum
Ileum
Caecum
Colon

Figure S4. Log10 normalised expression of major mouse proteins driving the energy metabolism pathways in the intestinal system during giardiasis, with respect to uninfected, cryptosporidiosis (positive control) and UPEC (negative control).

Pentose phosphate pathways and amino acid-sugar interconversions

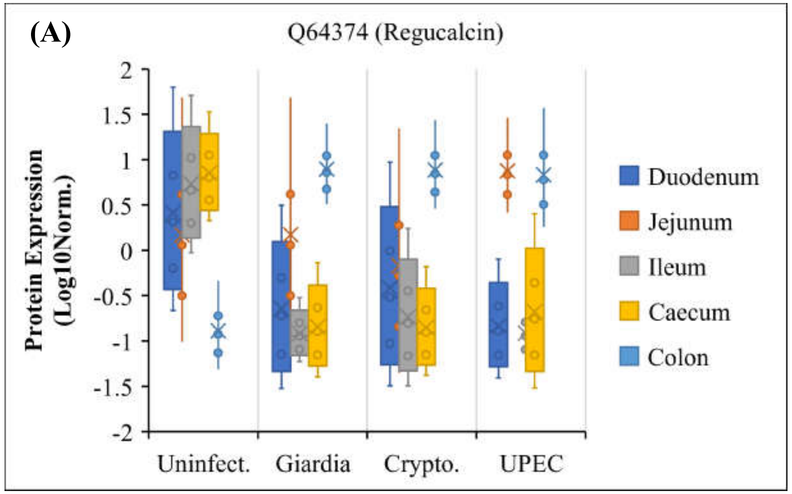

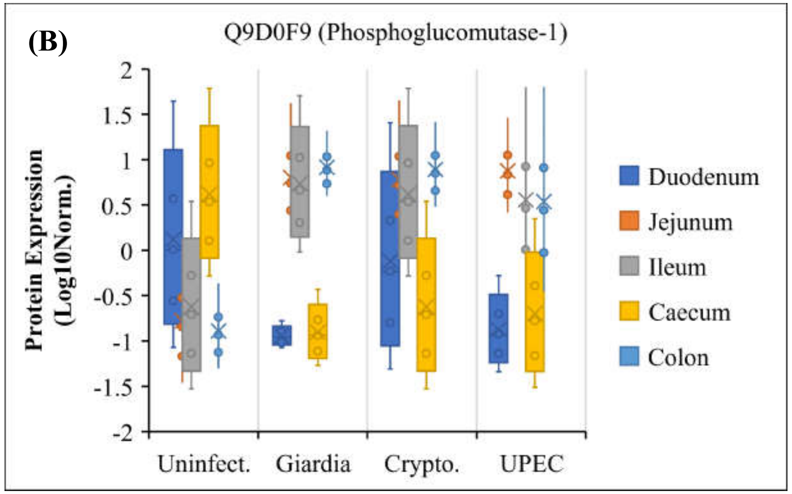

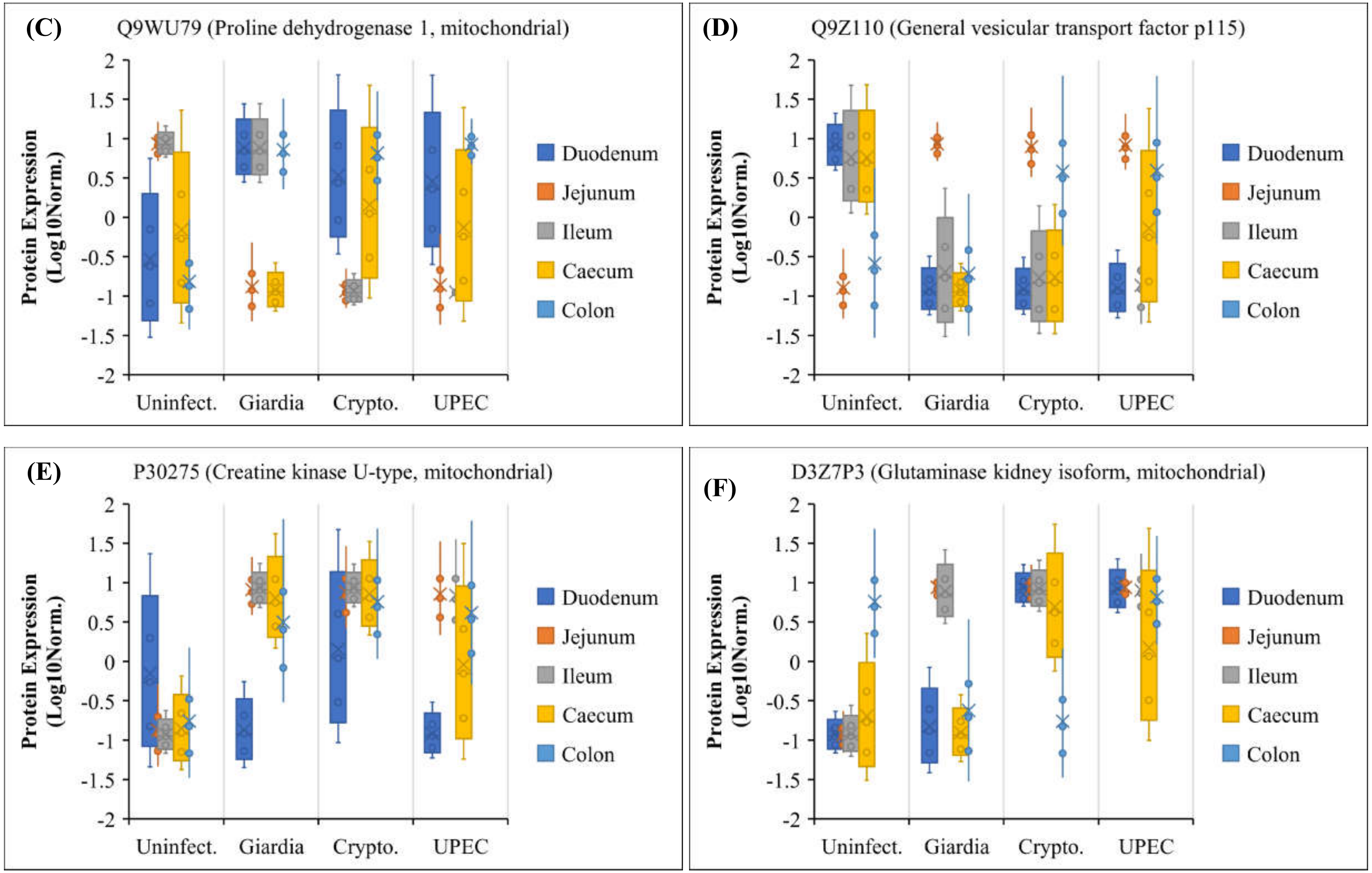
(C)
Q9WU79 (Proline dehydrogenase 1, mitochondrial)
Protein Expression (Log10Norm.)
2
1.5
1
0.5
0
-0.5
-1
-1.5
-2
Uninfect.
Giardia
Crypto.
UPEC
Duodenum
Jejunum
Ileum
Caecum
Colon
(D)
Q9Z110 (General vesicular transport factor p115)
(E)
P30275 (Creatine kinase U-type, mitochondrial)
(F)
D3Z7P3 (Glutaminase kidney isoform, mitochondrial)

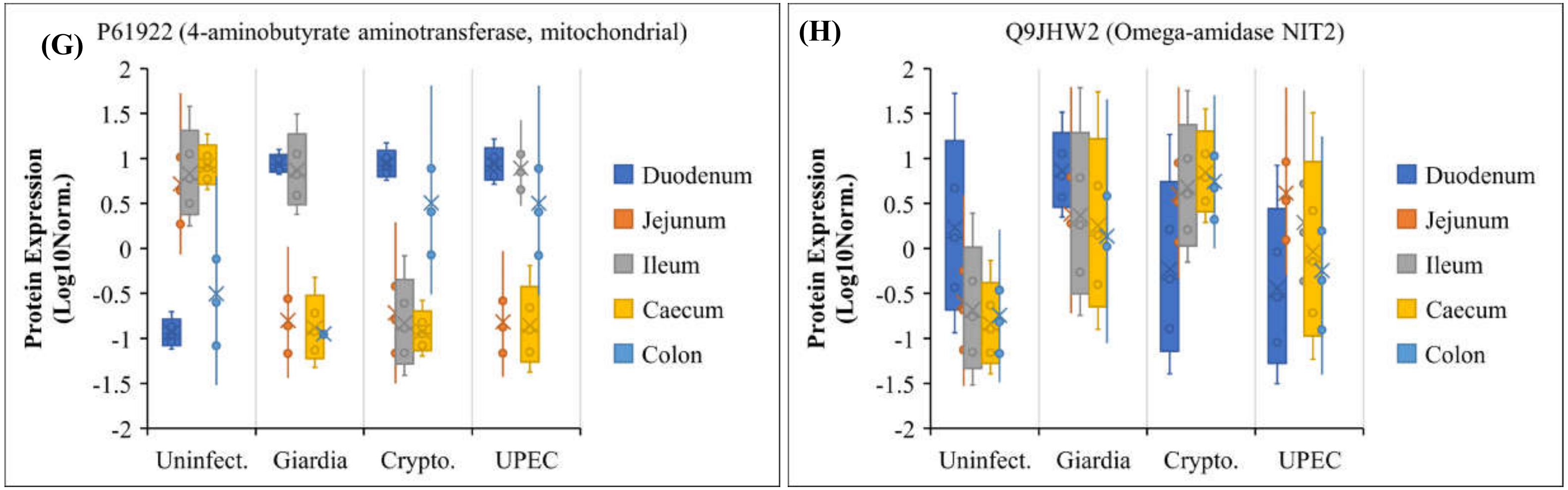

Figure S5. Log_{10} normalised expression of major mouse proteins driving the pentose phosphate pathways and amino acid-sugar interconversions in the intestinal system during giardiasis, with respect to uninfected, cryptosporidiosis (positive control) and UPEC (negative control).

Sulphur pathways (Glutathione metabolism, Cysteine and methionine metabolism)

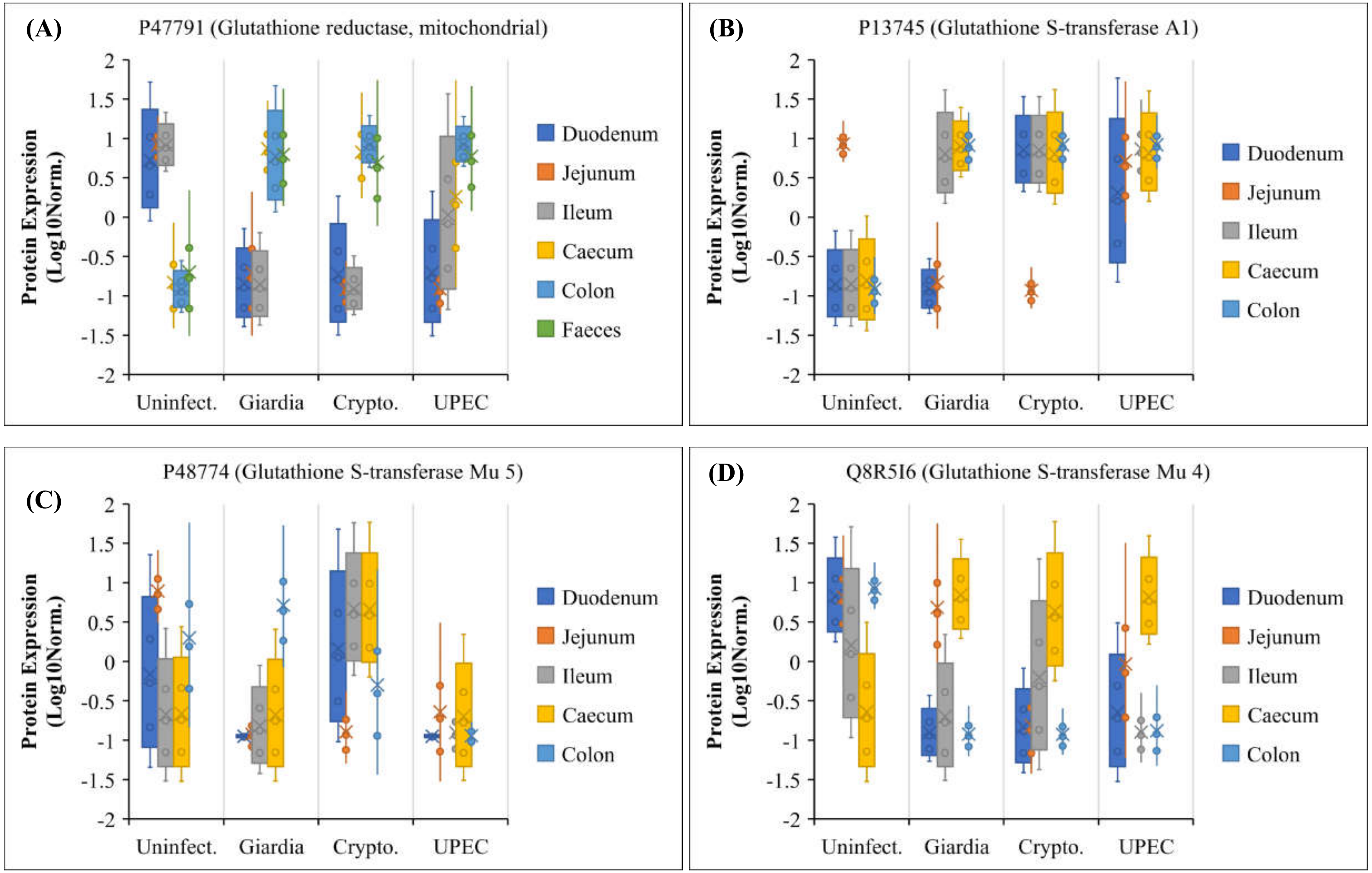

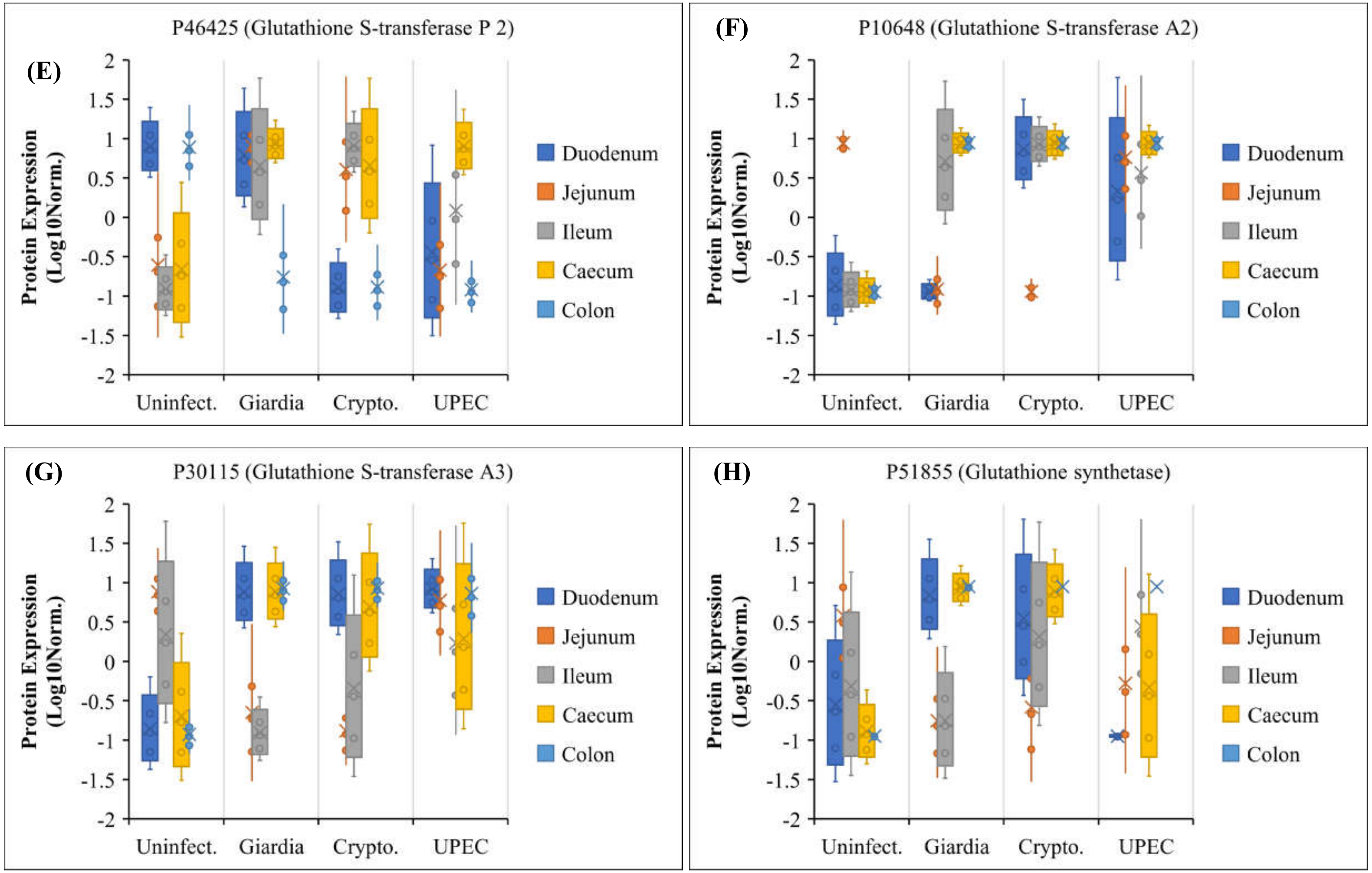
(E)
P46425 (Glutathione S-transferase P 2)
(F)
P10648 (Glutathione S-transferase A2)
(G)
P30115 (Glutathione S-transferase A3)
(H)
P51855 (Glutathione synthetase)
Protein Expression (Log10Norm.)
2
1.5
1
0.5
0
-0.5
-1
-1.5
-2
Uninfect.
Giardia
Crypto.
UPEC
Duodenum
Jejunum
Ileum
Caecum
Colon

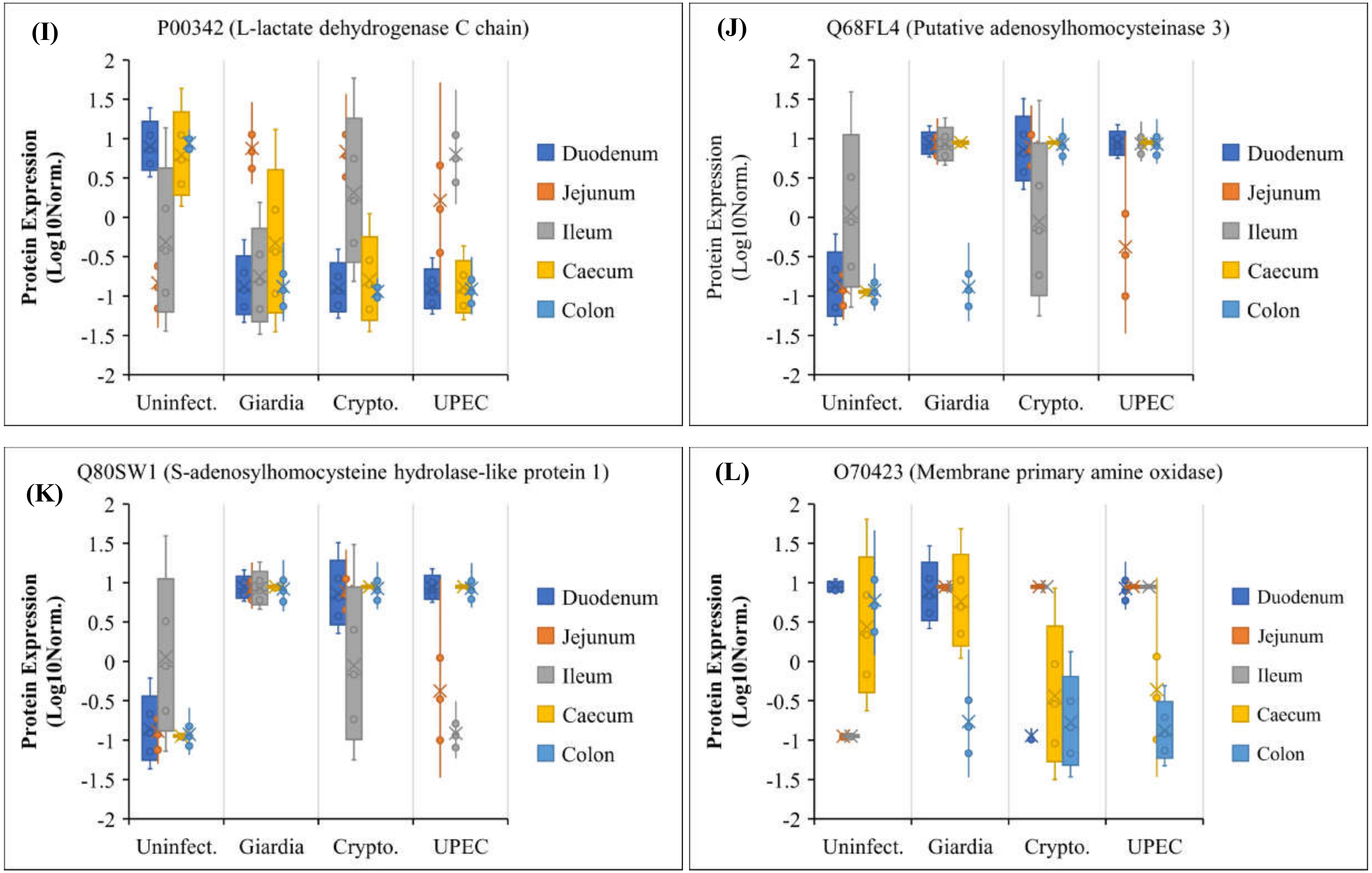

Figure S6. Log$_{10}$ normalised expression of major mouse proteins driving the sulphur metabolism pathways in the intestinal system during giardiasis, with respect to uninfected, cryptosporidiosis (positive control) and UPEC (negative control).

Nitrogen metabolism pathways

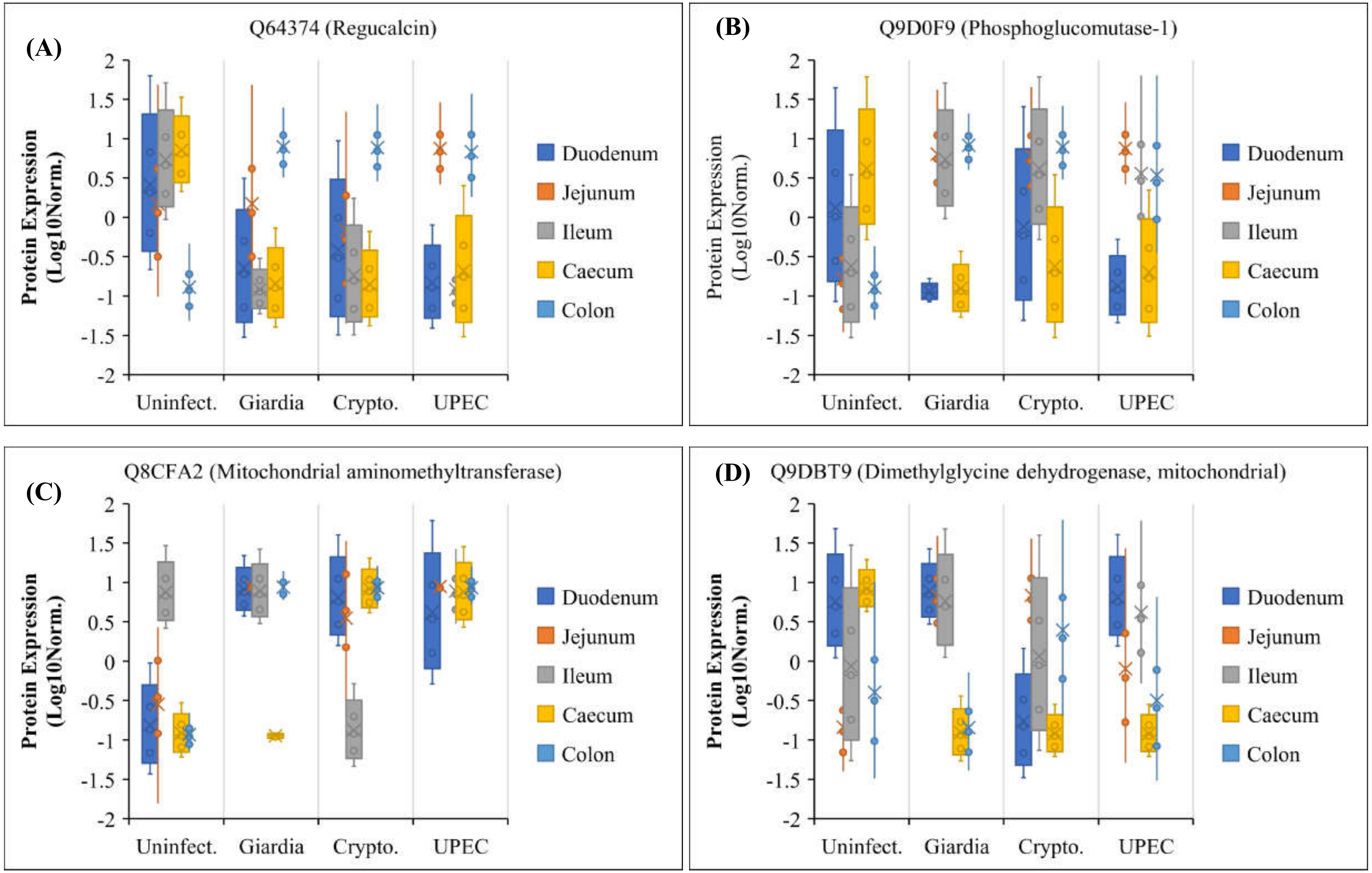

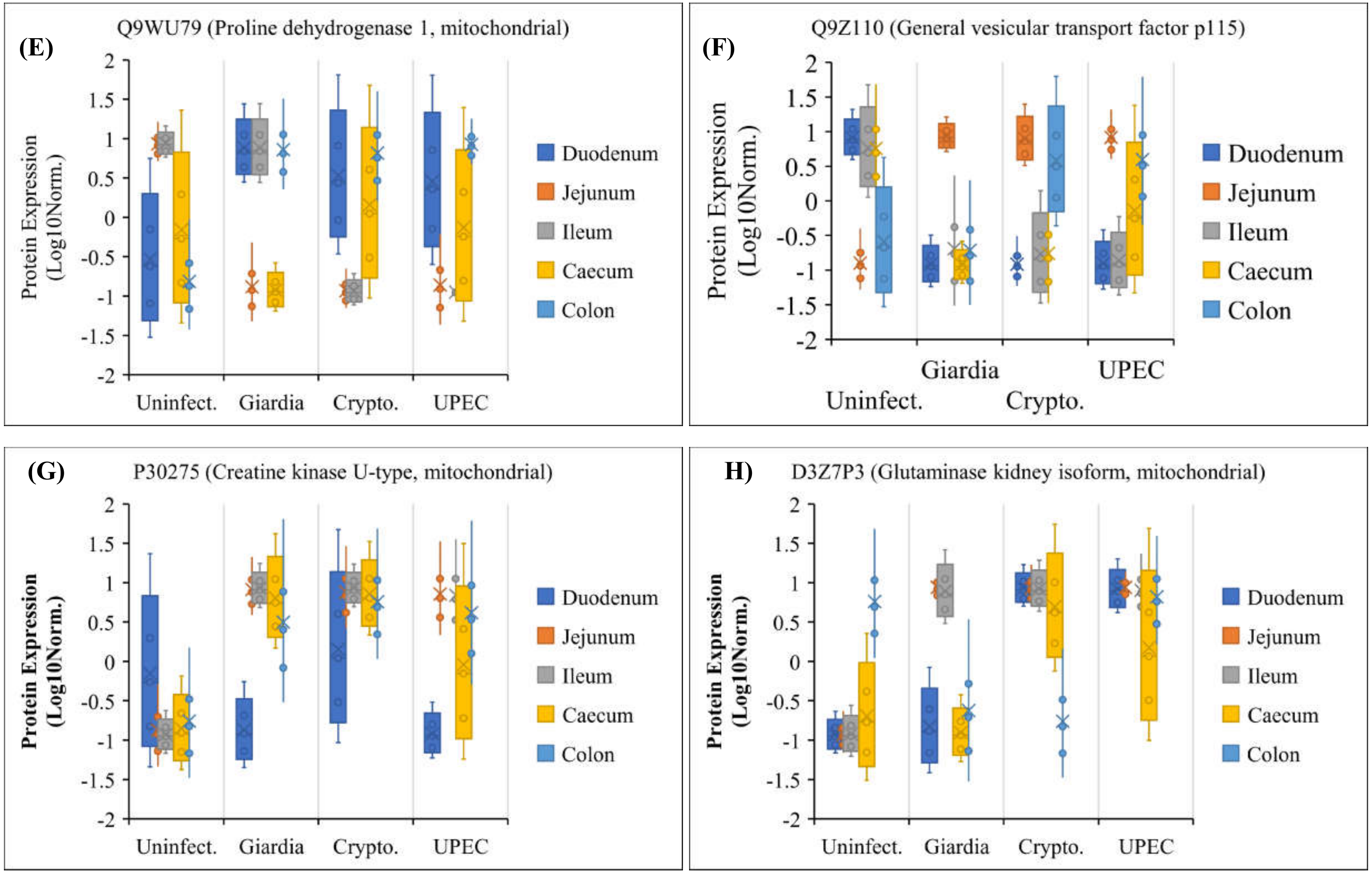

(E)
Q9WU79 (Proline dehydrogenase 1, mitochondrial)
Protein Expression (Log10Norm.)
2
1.5
1
0.5
0
-0.5
-1
-1.5
-2
Uninfect.
Giardia
Crypto.
UPEC
Duodenum
Jejunum
Ileum
Caecum
Colon
(F)
Q9Z110 (General vesicular transport factor p115)
Protein Expression (Log10Norm.)
Giardia
UPEC
Uninfect.
Crypto.
Duodenum
Jejunum
Ileum
Caecum
Colon
(G)
P30275 (Creatine kinase U-type, mitochondrial)
Protein Expression (Log10Norm.)
Uninfect.
Giardia
Crypto.
UPEC
Duodenum
Jejunum
Ileum
Caecum
Colon
H)
D3Z7P3 (Glutaminase kidney isoform, mitochondrial)
Protein Expression (Log10Norm.)
Uninfect.
Giardia
Crypto.
UPEC
Duodenum
Jejunum
Ileum
Caecum
Colon

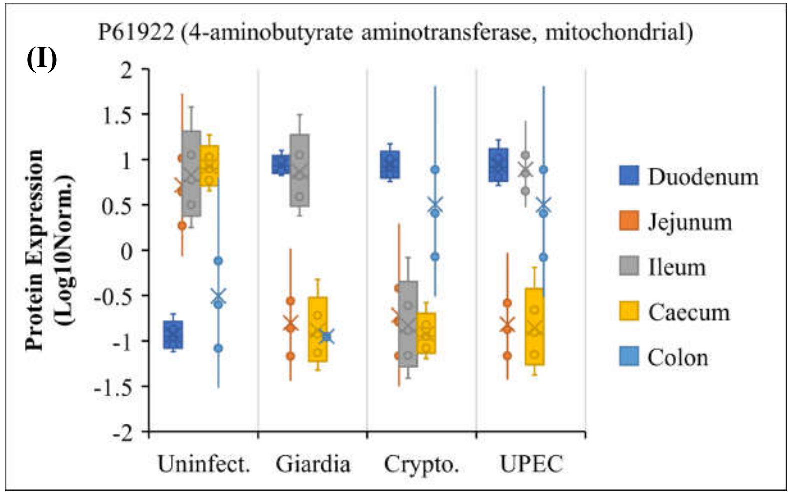

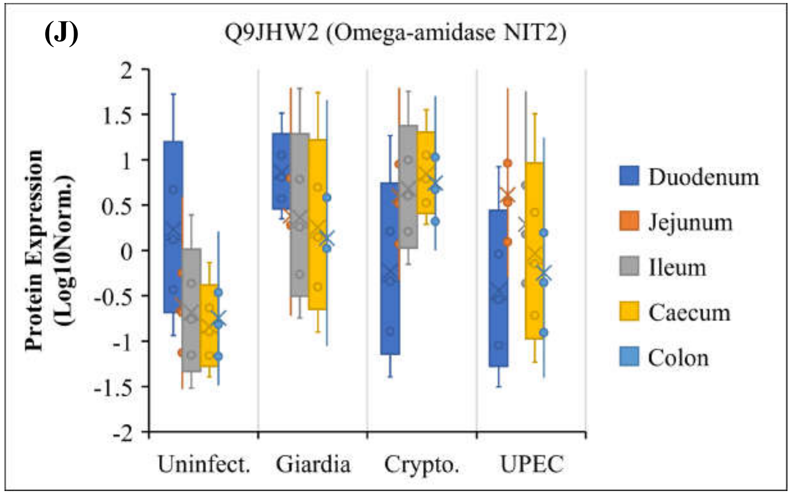

Figure S7. Log_{10} normalised expression of major mouse proteins driving the nitrogen metabolism pathways in the intestinal system during giardiasis, with respect to uninfected, cryptosporidiosis (positive control) and UPEC (negative control).

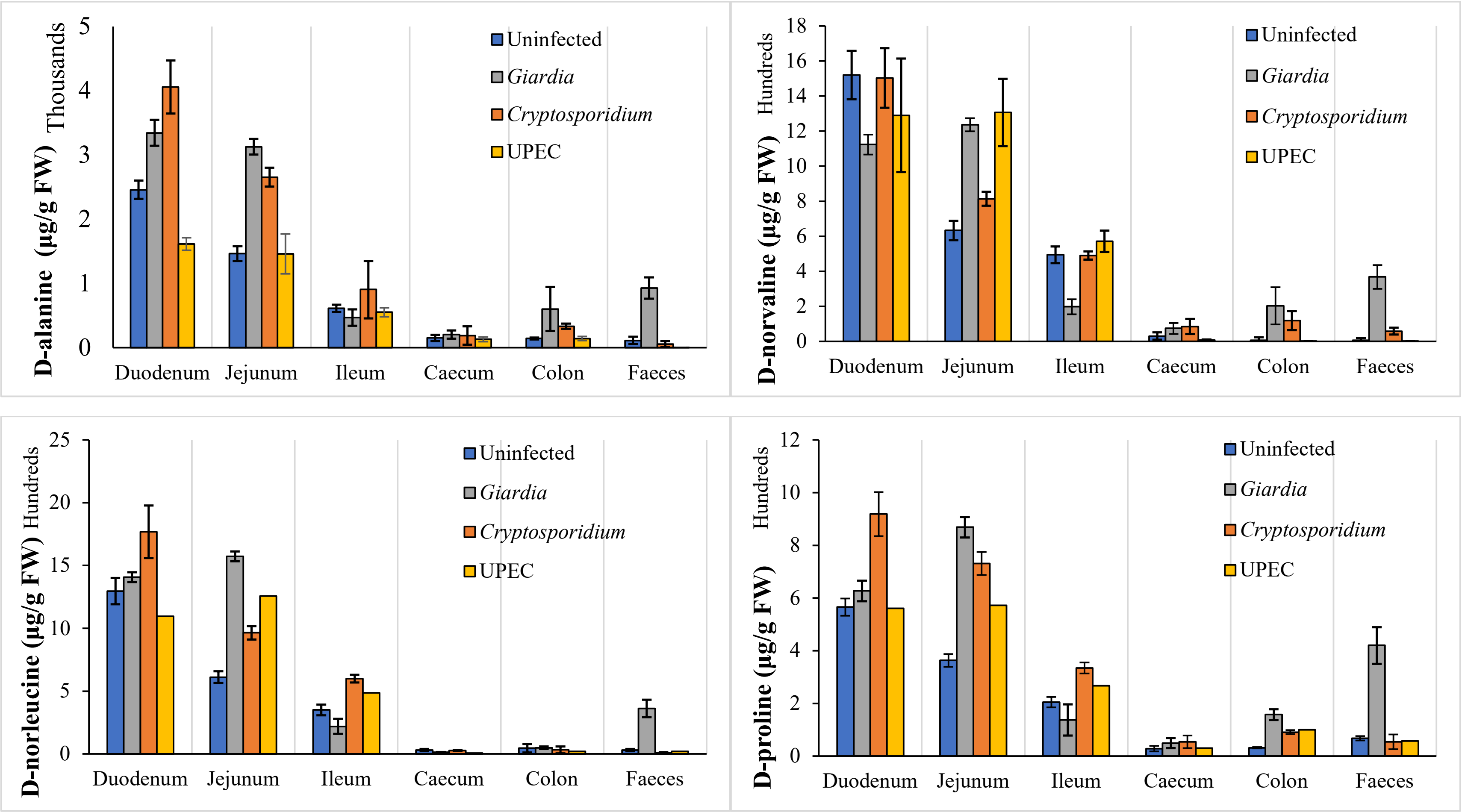

Figure S8. Accumulation of major D-amino acids throughout the mice gut during various infections. Error bars represent the standard deviation across experimental replicates (n = 5).

Table S2. Differential expression of major metabolic pathways in the mouse gut during cryptosporidiosis, giardiasis and UPEC infection in infected mice with respect to the uninfected mice. The values are represented as the normalised Log_2Fold changes, subjected to pareto scaling (n = 5). The terms refer to as C = *Cryptosporidium*, G = *Giardia*, U = UPEC.

Pathway	Metabolites↓	Duodenum			Jejunum			Ileum			Caecum			Colon		
	**Infections →**	**C**	**G**	**U**	**C**	**G**	**U**	**C**	**G**	**U**	**C**	**G**	**U**	**C**	**G**	**U**
Glutathione metabolism	Putrescine	-1.35	-0.29	-2.33	0.25	0.31	-0.53	2.01	1.87	0.67	-1.63	6.77	1.10	-0.91	0.67	-1.48
	Glycine	0.25	-0.34	0.11	0.74	1.45	1.53	3.21	3.10	2.06	1.06	-2.61	1.87	-3.22	-0.58	-1.26
	Dehydro-ascorbate	-1.13	-0.07	-1.10	0.44	-0.04	-0.49	-0.14	-0.14	0.33	11.11	10.41	1.06	0.16	1.20	-0.45
	Ascorbate	0.05	-0.76	-1.12	0.41	1.15	0.50	-2.18	0.83	-3.18	-1.34	0.29	-4.38	10.67	9.68	9.34
	Cysteine	-0.84	-0.36	-2.23	1.05	2.01	0.96	-0.29	1.25	3.80	-9.80	3.13	-7.09	0.63	1.27	1.16
	Glutathione	0.30	-0.24	-0.01	-0.08	-0.14	-0.55	2.89	2.35	1.77	-10.00	-10.19	1.52	0.40	2.40	1.46
	Glutamate	0.38	0.07	0.08	-1.24	-0.07	-1.40	0.46	0.49	0.23	0.06	-0.29	-0.45	0.31	-0.02	0.23
Glycolysis	Glucose	-0.05	-0.35	-0.56	0.29	0.34	-0.68	-0.89	-0.52	-0.78	0.16	-2.70	-0.21	1.05	1.33	0.002
	Lactate	0.05	-0.07	-0.23	0.23	0.13	0.03	0.60	0.52	-0.80	-12.73	-3.01	-1.57	-0.78	1.29	0.83
	Glucose-6P	0.59	0.40	0.37	-0.05	-1.01	-1.13	1.34	1.13	0.27	10.19	2.05	7.96	-1.69	-0.18	-1.39
	Glyceraldehyde-3P	-0.38	-0.25	-0.44	0.38	0.21	-0.28	0.94	-0.63	0.97	10.41	1.23	1.95	-0.32	1.56	0.07
	Pyruvate	-0.76	-0.50	-0.62	-1.34	-1.39	-1.59	7.19	7.44	8.77	1.30	6.29	7.99	-0.37	-1.63	-2.26
Pentose phosphate pathway	Glucose	-0.05	-0.35	-0.56	0.29	0.34	-0.68	-0.89	-0.52	-0.78	0.16	-2.70	-0.21	1.05	1.33	0.002
	Glucose-6P	0.59	0.40	0.37	-0.05	-1.01	-1.13	1.34	1.13	0.27	10.19	2.05	7.96	-1.69	-0.18	-1.39
	Glyceraldehyde-3P	-0.38	-0.25	-0.44	0.38	0.21	-0.28	0.94	-0.63	0.97	10.41	1.23	1.95	-0.32	1.56	0.07
	Pyruvate	-0.76	-0.50	-0.62	-1.34	-1.39	-1.59	7.19	7.44	8.77	1.30	6.29	7.99	-0.37	-1.63	-2.26
	Gluconolactone	-0.23	-0.56	-0.80	-0.30	-0.89	-1.47	2.07	1.86	0.64	11.70	0.69	7.70	1.82	1.32	1.01
	Ribose-5P	0.70	0.20	-0.27	0.78	0.36	0.57	1.04	0.45	0.15	-2.33	-0.48	-0.27	-0.68	0.48	-0.80
	Erythrose-4P	0.16	-0.14	-0.39	0.28	0.27	-0.63	1.61	1.15	-0.30	3.20	-1.85	5.75	0.08	0.75	1.00
	Glyceraldehyde	-0.38	-0.25	-0.44	0.38	0.21	-0.28	0.94	-0.63	0.97	10.41	1.23	1.95	-0.32	1.56	0.07
	Glycerate	0.07	-0.21	0.07	0.37	0.55	-0.10	0.54	1.14	1.42	-2.94	-0.56	0.97	-1.13	1.80	0.79
	Gluconate	-0.23	-0.56	-0.80	-0.30	-0.89	-1.47	2.07	1.86	0.64	11.70	0.69	7.70	1.82	1.32	1.01

Arginine and proline metabolism	Putrescine	-1.35	-0.29	-2.33	0.25	0.31	-0.53	2.01	1.87	0.67	-1.63	6.77	1.10	-0.91	0.67	-1.48
	5-aminovalerate	0.61	0.60	0.92	-0.91	-1.27	-2.19	1.74	1.59	1.46	3.78	3.13	1.38	0.33	2.06	1.96
	Urea	-0.38	-0.20	-0.35	0.92	0.52	0.43	1.03	0.66	0.33	-0.45	0.96	-9.27	0.54	1.61	-1.39
	Creatinine	0.12	-0.06	-0.15	-0.20	-0.06	-0.30	0.54	-0.32	0.84	-12.90	-4.52	7.85	-0.13	-0.53	-0.19
	5-Amino-pentanoate	-1.46	-0.31	-2.99	0.65	0.41	-0.54	0.40	0.74	-6.82	10.72	-3.99	-9.91	-1.27	-0.66	-1.59
	Pyruvate	-0.76	-0.50	-0.62	-1.34	-1.39	-1.59	7.19	7.44	8.77	1.30	6.29	7.99	-0.37	-1.63	-2.26
	Proline	0.20	0.58	1.11	-0.69	-1.09	-0.52	0.64	2.07	1.43	11.04	10.63	8.04	1.19	1.71	0.84
	Glutamate	0.38	0.07	0.08	-1.24	-0.07	-1.40	0.46	0.49	0.23	0.06	-0.29	-0.45	0.31	-0.02	0.23
Cysteine and methionine metabolism	Aspartate	1.44	0.33	1.35	0.95	0.46	-0.12	2.15	2.01	0.59	5.93	-5.59	5.17	-0.95	-0.16	-1.49
	Alanine	0.65	-0.20	-0.63	0.51	0.09	-1.11	-5.89	-2.71	0.30	0.49	5.25	4.69	0.69	0.93	-0.24
	Serine	0.35	-0.02	0.41	-0.27	0.02	-0.40	-4.38	-8.76	-0.42	-11.82	2.80	-6.96	-11.37	0.57	-0.80
	Methionine	0.48	-0.18	0.92	0.22	0.22	0.01	0.58	0.85	-0.60	-0.39	10.34	1.27	-0.66	1.29	0.50
	Cysteine	-0.84	-0.36	-2.23	1.05	2.01	0.96	-0.29	1.25	3.80	-9.80	3.13	-7.09	0.63	1.27	1.16
	Glutathione	0.30	-0.24	-0.01	-0.08	-0.14	-0.55	2.89	2.35	1.77	-10.00	-10.19	1.52	0.40	2.40	1.46
	Pyruvate	-0.76	-0.50	-0.62	-1.34	-1.39	-1.59	7.19	7.44	8.77	1.30	6.29	7.99	-0.37	-1.63	-2.26
Citrate Cycle	Succinate	0.19	-0.35	-0.31	0.48	-1.35	-0.67	0.91	1.74	1.42	10.93	0.66	1.05	0.37	2.09	1.80
	Fumarate	0.22	0.00	-0.34	0.18	0.00	-0.50	0.10	-0.96	-0.22	-2.71	-0.71	1.52	-0.97	1.43	0.13
	cis-Aconitate	0.16	0.82	0.51	-0.25	-0.24	-1.12	4.51	3.73	2.05	1.02	1.67	-0.99	1.09	2.97	2.37
	Citrate	-0.17	-0.20	-0.87	-0.38	0.80	0.11	2.33	1.94	2.41	-2.82	-2.51	-0.08	9.42	10.32	9.62
	Pyruvate	-0.76	-0.50	-0.62	-1.34	-1.39	-1.59	7.19	7.44	8.77	1.30	6.29	7.99	-0.37	-1.63	-2.26
	Oxoglutarate	0.19	0.47	-1.00	0.34	0.14	-0.01	0.30	0.27	0.38	-7.93	0.11	2.07	-0.82	0.36	-0.28
Alanine, aspartate and glutamate metabolism	Aspartate	1.44	0.33	1.35	0.95	0.46	-0.12	2.15	2.01	0.59	5.93	-5.59	5.17	-0.95	-0.16	-1.49
	Alanine	0.65	-0.20	-0.63	0.51	0.09	-1.11	-5.89	-2.71	0.30	0.49	5.25	4.69	0.69	0.93	-0.24
	Succinate	0.19	-0.35	-0.31	0.48	-1.35	-0.67	0.91	1.74	1.42	10.93	0.66	1.05	0.37	2.09	1.80
	Fumarate	0.22	0.00	-0.34	0.18	0.00	-0.50	0.10	-0.96	-0.22	-2.71	-0.71	1.52	-0.97	1.43	0.13
	Glutamine	-0.32	-1.47	-0.28	-1.14	-0.67	-1.44	0.90	2.02	0.43	-0.50	-0.68	8.56	6.34	6.95	7.55
	Asparagine	-0.18	-0.32	-1.40	-0.20	0.36	0.24	0.92	0.89	0.34	-2.92	-0.11	3.37	0.20	1.73	0.98
	Citrate	-0.17	-0.20	-0.87	-0.38	0.80	0.11	2.33	1.94	2.41	-2.82	-2.51	-0.08	9.42	10.32	9.62
	Pyruvate	-0.76	-0.50	-0.62	-1.34	-1.39	-1.59	7.19	7.44	8.77	1.30	6.29	7.99	-0.37	-1.63	-2.26

	Oxoglutarate	0.19	0.47	-1.00	0.34	0.14	-0.01	0.30	0.27	0.38	-7.93	0.11	2.07	-0.82	0.36	-0.28
	Glutamate	0.38	0.07	0.08	-1.24	-0.07	-1.40	0.46	0.49	0.23	0.06	-0.29	-0.45	0.31	-0.02	0.23
Propanoate and Butanoate metabolism	Succinate	0.19	-0.35	-0.31	0.48	-1.35	-0.67	0.91	1.74	1.42	10.93	0.66	1.05	0.37	2.09	1.80
	Lactate	0.05	-0.07	-0.23	0.23	0.13	0.03	0.60	0.52	-0.80	-12.73	-3.01	-1.57	-0.78	1.29	0.83
	Methylmalonate	0.62	0.71	0.27	-0.54	-0.28	-0.42	1.50	1.45	0.49	-1.50	9.57	-6.84	-2.05	1.65	-0.01
	2-hydroxy-butyrate	-1.25	0.78	-0.26	-1.80	-0.16	-0.31	-0.42	0.06	-1.58	-0.68	-1.23	1.24	1.46	1.54	0.45
	Fumarate	0.22	0.00	-0.34	0.18	0.00	-0.50	0.10	-0.96	-0.22	-2.71	-0.71	1.52	-0.97	1.43	0.13
	Pyruvate	-0.76	-0.50	-0.62	-1.34	-1.39	-1.59	7.19	7.44	8.77	1.30	6.29	7.99	-0.37	-1.63	-2.26
	Oxoglutarate	0.19	0.47	-1.00	0.34	0.14	-0.01	0.30	0.27	0.38	-7.93	0.11	2.07	-0.82	0.36	-0.28
	Glutamate	0.38	0.07	0.08	-1.24	-0.07	-1.40	0.46	0.49	0.23	0.06	-0.29	-0.45	0.31	-0.02	0.23
	D-malate	0.32	0.12	-0.26	0.37	0.39	-0.50	1.34	1.24	0.21	-6.09	-6.86	0.00	-0.21	-1.27	-0.85
	Maleate	0.32	0.12	-0.26	0.29	0.39	-0.57	1.47	1.15	0.08	1.71	1.58	0.87	-1.10	0.01	0.43

Table S3. Differential expression of major metabolic pathways in the mouse extra-gut organs during cryptosporidiosis, giardiasis and UPEC infection in infected mice with respect to the uninfected mice. The values are represented as the normalised Log_2Fold changes, subjected to pareto scaling (n = 5).

**Pathway**	**Serum**			**Liver**		
	Crypto.	Giardia	UPEC	Crypto.	Giardia	UPEC
Glycolysis	-0.51	1.27	-0.76	0.85	1.08	-1.92
Glutathione metabolism	-0.32	3.68	-3.36	0.26	-2.68	2.42
Purine metabolism	0.81	-1.24	0.42	1.22	1.37	-2.59
Gly, Ser, Thr metabolism	0.91	-2.21	1.30	1.27	-0.39	-0.89
Glyoxylate, dicarboxylate metabolism	0.41	-1.35	0.95	1.34	0.12	-1.46
Arginine, proline metabolism	-1.89	2.33	-1.14	0.82	0.55	-1.37
Propanoate metabolism	0.49	1.01	-1.50	1.03	-1.85	0.81
Cysteine, methionine metabolism	0.79	1.05	1.85	1.93	-1.73	-0.21
Citrate cycle	-0.94	1.18	-0.24	-0.21	0.86	-0.65
Ala, Asp, Glu metabolism	-1.21	0.20	1.00	1.22	-0.44	-0.78
Butanoate metabolism	0.29	-0.52	0.24	0.15	-0.60	0.45
Pentose phosphate pathway	-0.34	0.73	-0.39	0.66	-0.90	0.24
Phenylalanine metabolism	0.89	-2.39	1.51	-0.46	0.05	0.41
Val, Leucine, Isoleucine biosynthesis	0.42	1.22	-1.64	-0.07	1.09	-1.02
Pyruvate metabolism	0.46	-0.94	0.48	0.71	1.27	-1.97

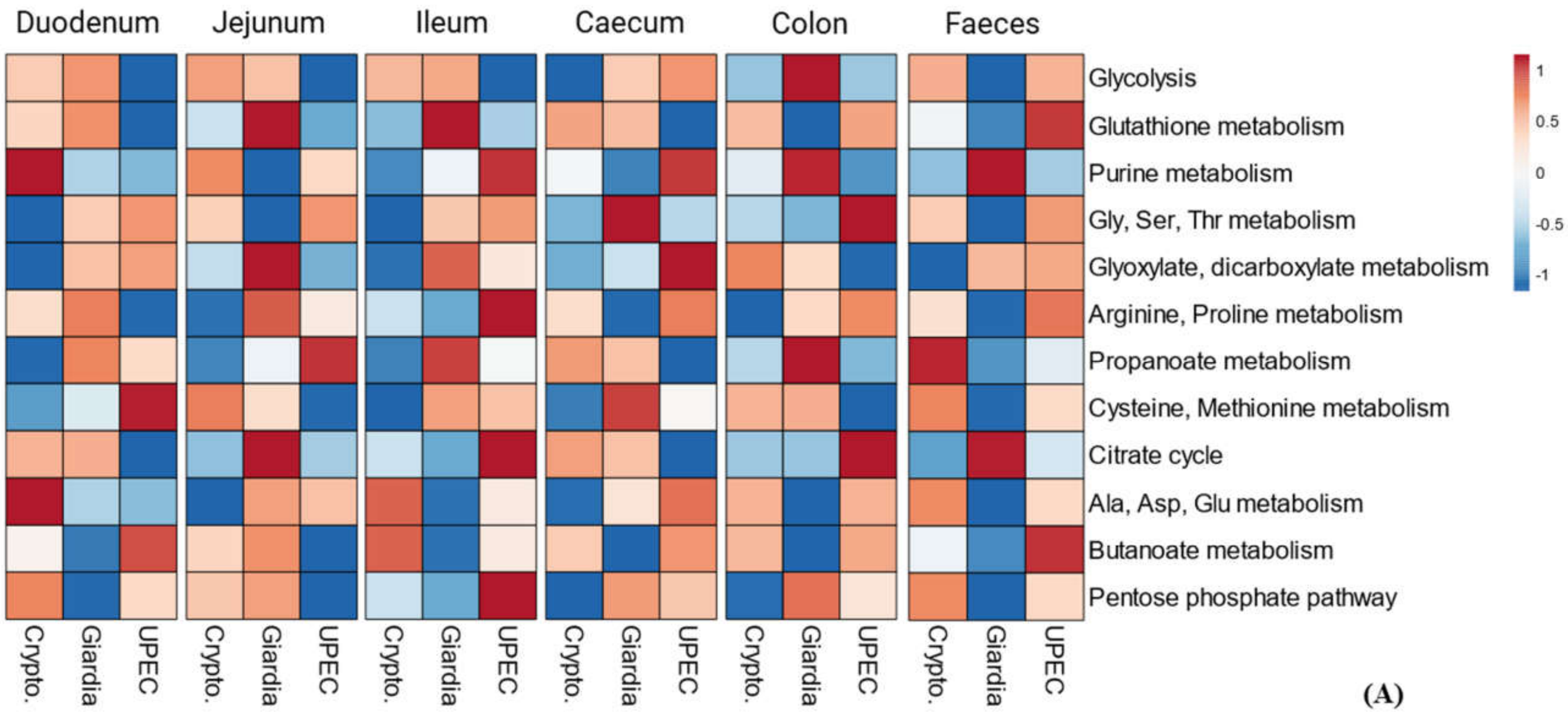

Duodenum
Jejunum
Ileum
Caecum
Colon
Faeces
Glycolysis
Glutathione metabolism
Purine metabolism
Gly, Ser, Thr metabolism
Glyoxylate, dicarboxylate metabolism
Arginine, Proline metabolism
Propanoate metabolism
Cysteine, Methionine metabolism
Citrate cycle
Ala, Asp, Glu metabolism
Butanoate metabolism
Pentose phosphate pathway
Crypto.
Giardia
UPEC
1
0.5
0
-0.5
-1

(A)

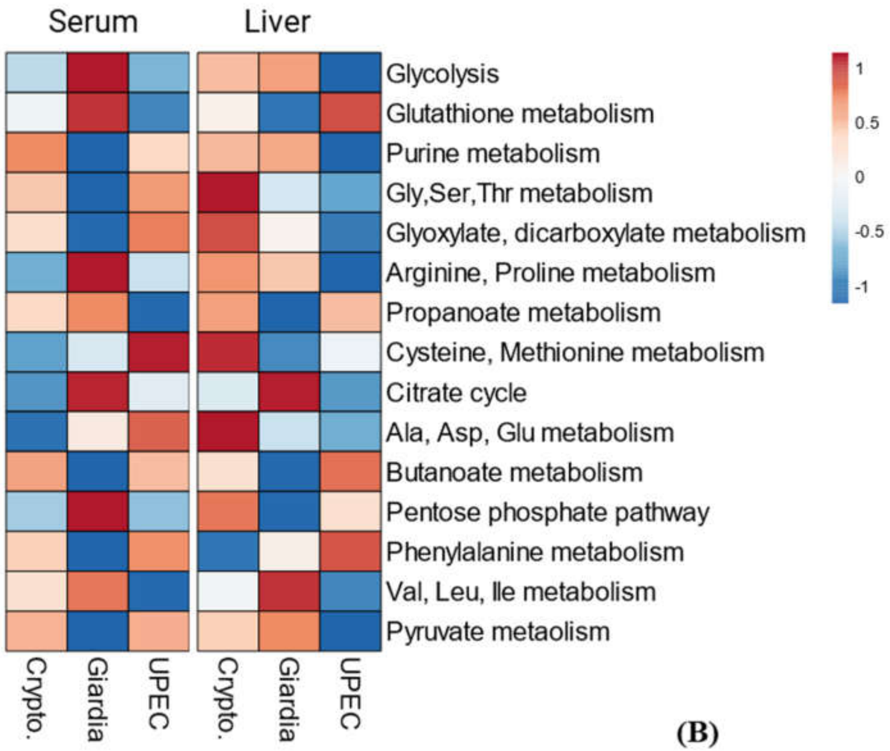

(B)

Figure S9. The heatmap represents relative upregulation (red) and downregulation (blue) of major metabolic pathways in (A) Intestine and faeces and, (B) Serum and liver, during cryptosporidiosis, giardiasis and UPEC infections in the infected mice with respect to the uninfected mice. The individual boxes indicate the relative impact as determined by the integrated proteomics-metabolomics analysis. The heatmaps were designed using Clustvis web tool.

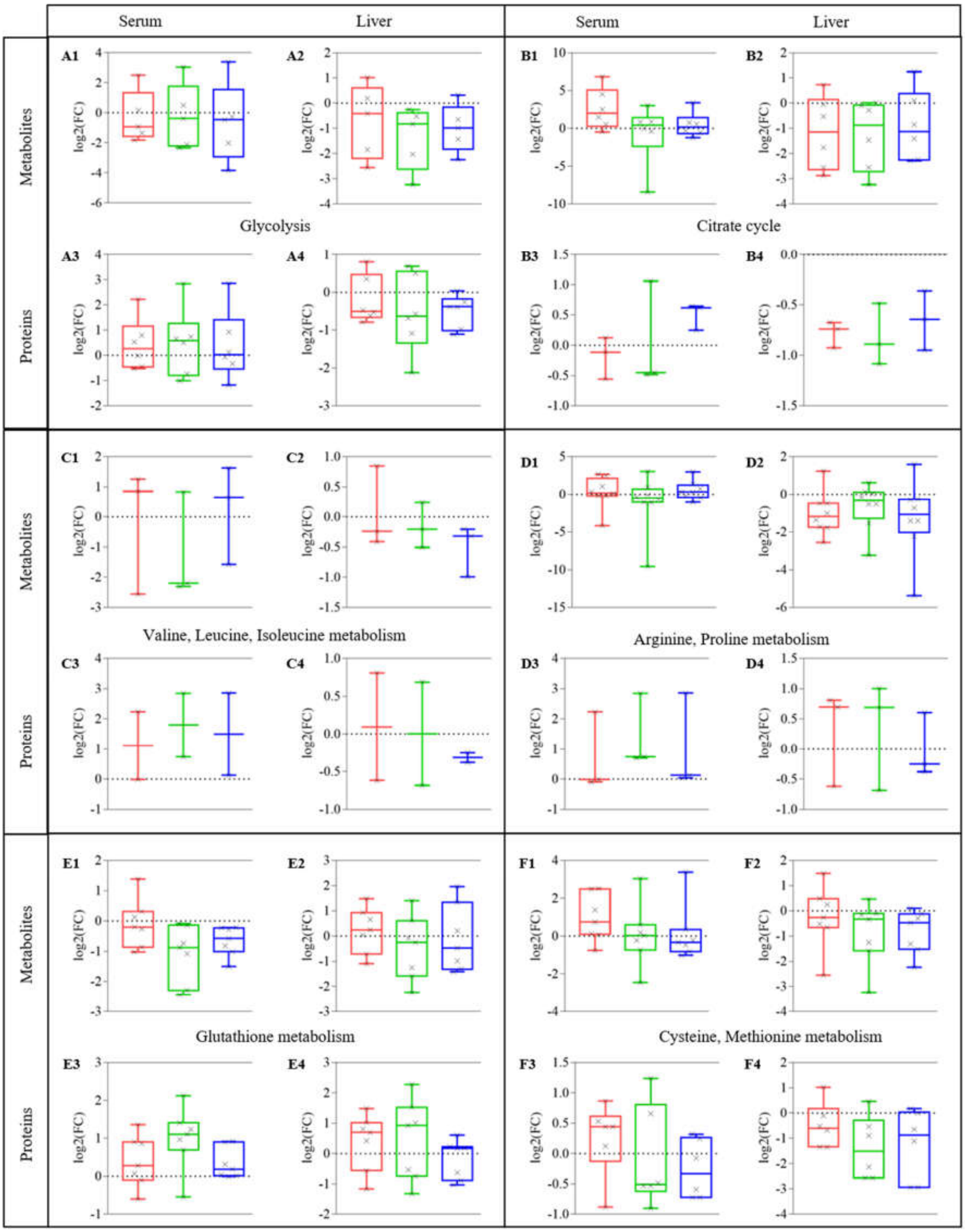

Figure S10. The comparative metabolite and protein expressions driving the key metabolic pathways in serum and liver during cryptosporidiosis (red), giardiasis (green) and UPEC (blue). The individual metabolites and proteins contributing to these expressions are presented in Table S4 and S5.

Table S4. Major metabolites contributing to the most significant metabolic pathways in serum and liver during various infections with respect to the uninfected mice.

**Pathway**	**Metabolites**	**Serum (Log$_2$ Fold change)**			**Liver (Log$_2$ Fold change)**		
		Crypto	Giardia	UPEC	Crypto	Giardia	UPEC
Glycolysis	Glucose	-0.93	-0.39	-0.47	1.02	-0.26	-1.42
	Lactate	-1.35	0.49	-0.27	0.19	-0.52	0.33
	Glucose-6P	-1.82	-2.12	-2.02	-1.84	-2.02	-0.98
	Glyceraldehyde-3P	0.16	-2.34	-3.84	-0.41	-0.83	-0.65
	Pyruvate	2.49	3.04	3.37	-2.55	-3.23	-2.24
Citrate cycle	Succinate	4.51	0.88	0.57	-0.04	-0.28	-0.85
	Fumarate	6.83	0.00	-1.23	0.72	0.02	1.25
	Cis-acotinate	0.52	-8.41	-0.29	-1.76	-2.55	-2.28
	Citrate	1.48	0.83	0.76	-2.87	-1.46	-1.40
	Pyruvate	2.49	3.04	3.37	-2.55	-3.23	-2.24
	Oxoglutarate	-0.46	-0.36	-0.53	-0.53	-0.10	0.10
Valine, leucine, isoleucine metabolism	Valine	-2.56	-2.30	1.62	0.84	0.24	-0.21
	Methylmalonate	0.85	0.83	0.64	-0.24	-0.51	-0.99
	Isoleucine	1.25	-2.20	-1.58	-0.41	-0.20	-0.32
Arginine and proline metabolism	Putrescine	0.31	-1.08	0.28	-1.01	0.61	-1.41
	2-oxo-4-hydroxy-5-aminovalerate	-4.17	-9.55	-0.18	-0.49	-1.54	-1.41
	Urea	-0.19	-1.06	-1.08	-1.36	-0.52	-0.72
	Creatinine	0.09	-0.10	0.36	1.24	0.14	1.60
	5-aminopentanoate	-0.14	-0.64	-0.46	-1.73	-0.52	-0.29
	Pyruvate	2.49	3.04	3.37	-2.55	-3.23	-2.24
	Proline	2.68	0.95	0.76	-1.77	0.03	-5.38
	Glutamate	1.08	-0.38	1.40	-0.49	-0.17	-0.26
Glutathione metabolism	Putrescine	0.31	-1.08	-0.28	-1.10	0.61	-1.41
	Glycine	-0.26	-2.29	-1.51	0.93	-2.24	0.21
	Dehydroascorbate	-1.02	-0.09	-0.22	0.04	-0.08	-0.99
	Ascorbate	-0.86	-0.88	-0.58	0.66	1.41	1.96
	Cysteine	1.38	-2.45	-1.02	1.48	-1.59	-1.31
	Glutathione	0.13	-0.74	-0.82	0.25	-1.26	-0.48
	Glutamate	1.08	-0.38	1.40	-0.49	-0.17	-0.26
Cysteine, Methionine metabolism	Aspartate	2.51	0.60	-0.46	-0.52	-0.10	0.10
	Alanine	0.09	0.18	0.35	-0.26	-0.16	-0.12
	Serine	-0.76	0.02	-0.22	0.49	0.47	-0.30
	Methionine	0.74	-0.22	-0.33	-0.66	-0.33	-1.52
	Cysteine	1.38	-2.45	-1.02	1.48	-1.59	-1.31
	Glutathione	0.13	-0.74	-0.82	0.25	-1.26	-0.48
	Pyruvate	2.49	3.04	3.37	-2.55	-3.23	-2.24

Table S5. Major protein expressions driving the metabolic pathways in serum and liver during various infections with respect to the uninfected mice.

Pathway	Serum (Log$_2$ Fold change)				Liver (Log$_2$ Fold change)			
	**Protein (Uniprot ID)**	**Crypto**	**Giardia**	**UPEC**	**Protein (Uniprot ID)**	**Crypto**	**Giardia**	**UPEC**
Glycolysis	O70250	1.65	0.99	2.30	P05064	0.35	0.51	0.04
	P15327	0.76	0.76	1.38	P47740	0.81	0.69	-0.25
	P05064	-0.45	-0.74	-0.33	Q9D0F9	-0.41	-0.96	-1.03
	P47740	-0.01	0.74	0.12	Q9DBJ1	-0.47	-0.57	-0.38
	Q9DBJ1	0.79	0.50	0.93	P16125	-0.52	-2.13	-1.11
	P16125	0.53	0.66	-0.07	Q9DBF1	-0.51	-0.45	-0.63
	O08749	-0.92	-0.53	-0.78	P00329	-0.72	-0.55	-0.94
	P16858	-0.64	-0.65	-0.74	P17182	-0.29	-0.61	-0.43
	P09041	0.02	-0.60	0.04	P28474	-0.79	-1.08	-0.98
	Q9JII6	-0.65	0.60	0.72				
	P28474	-0.52	-1.01	-1.18				
	P06745	-0.17	0.63	-0.27				
	Q9JLJ2	2.23	2.85	2.86				
Citrate cycle	P14152	0.12	-0.49	0.25	P54071	-0.77	-0.88	-0.93
	Q60597	0.06	-0.94	-0.08	P08249	-0.41	-0.58	-0.25
	Q9WUM5	-0.11	-0.45	0.62	P14152	-0.68	-0.89	-0.64
	O08749	-0.92	-0.53	-0.79	Q9D6R2	0.62	1.25	0.29
	Q8K2B3	-0.52	0.59	0.19	P28271	0.04	-0.54	-0.86
	Q91V92	-0.56	1.06	0.65	Q9WUM5	-0.74	-1.09	-0.36
	O88844	0.04	0.57	0.08	P97807	-0.11	-0.59	-0.32
					Q91V92	-0.93	-0.48	-0.95
					Q9Z2I9	-0.85	-0.85	-0.86
Valine, Leucine, Isoleucine metabolism	Q9JHI5	-0.21	0.58	-0.48	Q91ZA3	-0.26	-0.76	-0.35
	P47740	-0.01	0.74	0.12	G3X982	-1.24	-1.22	-1.05
	P50136	-2.52	-1.77	-1.31	P47740	0.81	0.69	-0.25
	O08749	-0.92	-0.53	-0.79	P38060	-0.22	-0.68	-0.01
	Q8QZS1	-0.97	-0.47	-2.46	Q9DBF1	-0.51	-0.45	-0.63
	Q99MR8	1.71	1.72	0.87	O08756	0.21	0.75	-0.18
	Q8QZT1	0.11	-0.67	0.07	Q8BMS1	-0.60	-0.75	-0.76
	Q8VCH0	-0.28	-0.58	0.59	P61922	-0.53	-0.64	-0.55
	Q9JLJ2	2.23	2.85	2.86	Q8BH95	-0.37	-0.52	-0.13
					P47738	-0.38	-0.59	-0.67
					Q61425	-0.41	-0.46	-0.30
					Q9JLJ2	-0.62	-0.68	-0.38
					Q921H8	-0.83	-0.75	-0.52
Arginine, proline metabolism	P47740	-0.01	0.74	0.12	P47740	0.81	0.69	-0.25
	A2AS89	-0.16	2.57	0.22	Q61176	1.37	1.76	1.07
	O08691	0.89	1.05	-0.26	Q9Z110	-1.13	-0.46	-0.69

	Q9D1A2	0.08	-0.56	1.18	Q9DBF1	-0.51	-0.45	-0.63
	P30275	0.16	1.57	0.32	Q9DCU9	0.54	0.44	-0.97
	Q9CPY7	-0.10	0.70	0.04	P29758	-0.32	-0.63	-0.39
	P05201	0.28	1.62	0.99	P07310	0.39	0.90	1.47
	Q9JLJ2	2.23	2.85	2.86	Q9CPY7	0.69	1.00	0.60
					P05202	-0.91	-0.75	-0.87
					P47738	-0.38	-0.59	-0.67
					Q9JLJ2	-0.62	-0.68	-0.38
Glutathione metabolism	Q64471	0.07	1.11	0.19	P47791	0.42	0.93	0.18
	O70325	-0.73	-0.83	-0.08	P54071	-0.77	-0.88	-0.93
	Q9CPY7	1.36	2.13	0.92	P10649	-0.60	-0.91	-0.91
	P21765	0.28	1.41	-0.01	Q8R5I6	-1.17	-1.33	-0.89
	P97494	-0.59	-0.55	0.02	P19639	-1.46	-2.21	-0.76
	P97449	0.21	0.78	-0.76	O35660	-0.35	-1.77	0.27
	O88844	-0.10	0.70	0.04	Q9DCD0	-0.14	0.71	0.32
	P30115	0.91	0.97	0.90	P46412	0.64	1.37	0.37
	P51855	0.87	1.24	0.32	P11352	0.80	1.52	-1.04
	P13745	1.04	0.71	0.84	Q64471	-0.56	-0.75	-0.63
	Q64471	0.04	0.57	0.08	Q91VS7	0.17	-1.31	-0.36
	O70325	0.15	1.79	-0.16	Q9DCM2	-0.77	-0.54	-1.23
	Q9CPY7	1.72	1.40	0.14	Q80W21	-0.35	-1.77	0.27
	P21765	0.95	4.26	-1.67	P15626	-0.29	-0.65	-0.88
					Q9CPY7	0.69	1.00	0.60
					Q8K010	0.74	0.73	-0.07
					P21765	1.48	2.28	0.23
					P97494	1.02	-0.53	0.17
					Q60928	1.25	1.12	0.71
Cysteine, Methionine metabolism	P47791	0.44	-0.53	-0.72	Q91WT9	-0.79	-0.70	-0.92
	Q9CQU0	-0.17	-0.47	-0.03	P06151	-0.35	-0.69	-0.91
	Q8R5I6	0.53	0.66	-0.07	Q8VBT2	-0.50	-1.14	-0.73
	P11352	0.12	-0.49	0.25	P16125	-0.52	-2.13	-1.11
	Q80SW1	1.29	1.50	-0.14	P08249	-0.41	-0.58	-0.25
	Q91X83	0.44	-0.65	0.92	P14152	-0.68	-0.89	-0.64
	P16125	0.44	-0.53	-0.72	Q80SW1	-1.34	-2.56	-2.94
	P14152	0.28	1.62	0.99	Q68FL4	-1.34	-2.56	-2.94
	P52196	-0.88	-0.90	-0.59	P05202	-0.91	-0.75	-0.87
	Q3THS6	0.87	1.24	0.32	Q99J99	-0.10	0.46	0.00
	Q68FL4	1.72	1.40	0.14	P97494	1.02	-0.53	0.17
					P50247	-0.60	-0.88	-0.68
					Q8VCN5	-0.67	-0.68	-0.42

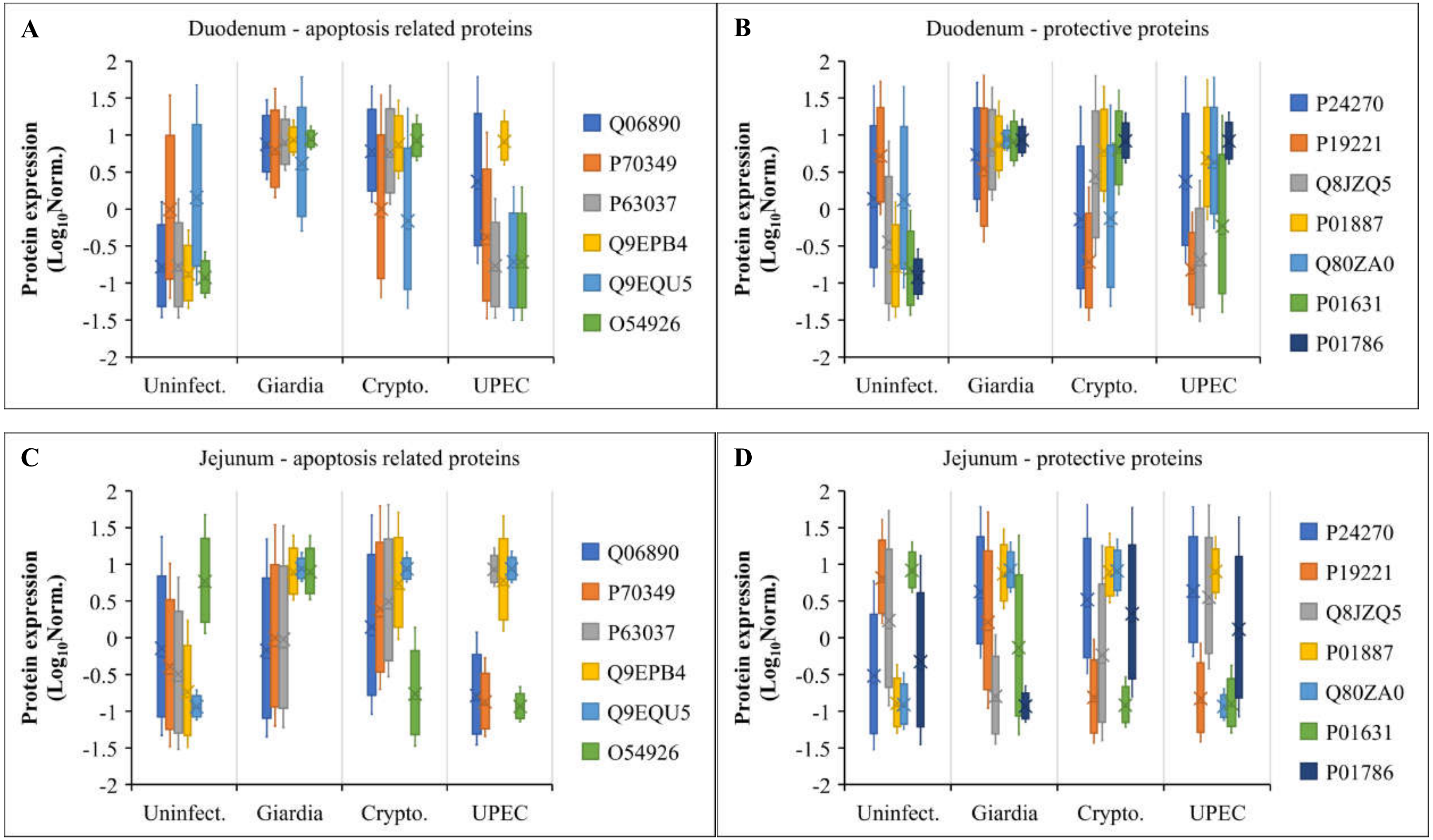
A
Duodenum - apoptosis related proteins
B
Duodenum - protective proteins
C
Jejunum - apoptosis related proteins
D
Jejunum - protective proteins
Protein expression (Log10Norm.)
2
1.5
1
0.5
0
-0.5
-1
-1.5
-2
Uninfect.
Giardia
Crypto.
UPEC
Q06890
P70349
P63037
Q9EPB4
Q9EQU5
O54926
P24270
P19221
Q8JZQ5
P01887
Q80ZA0
P01631
P01786

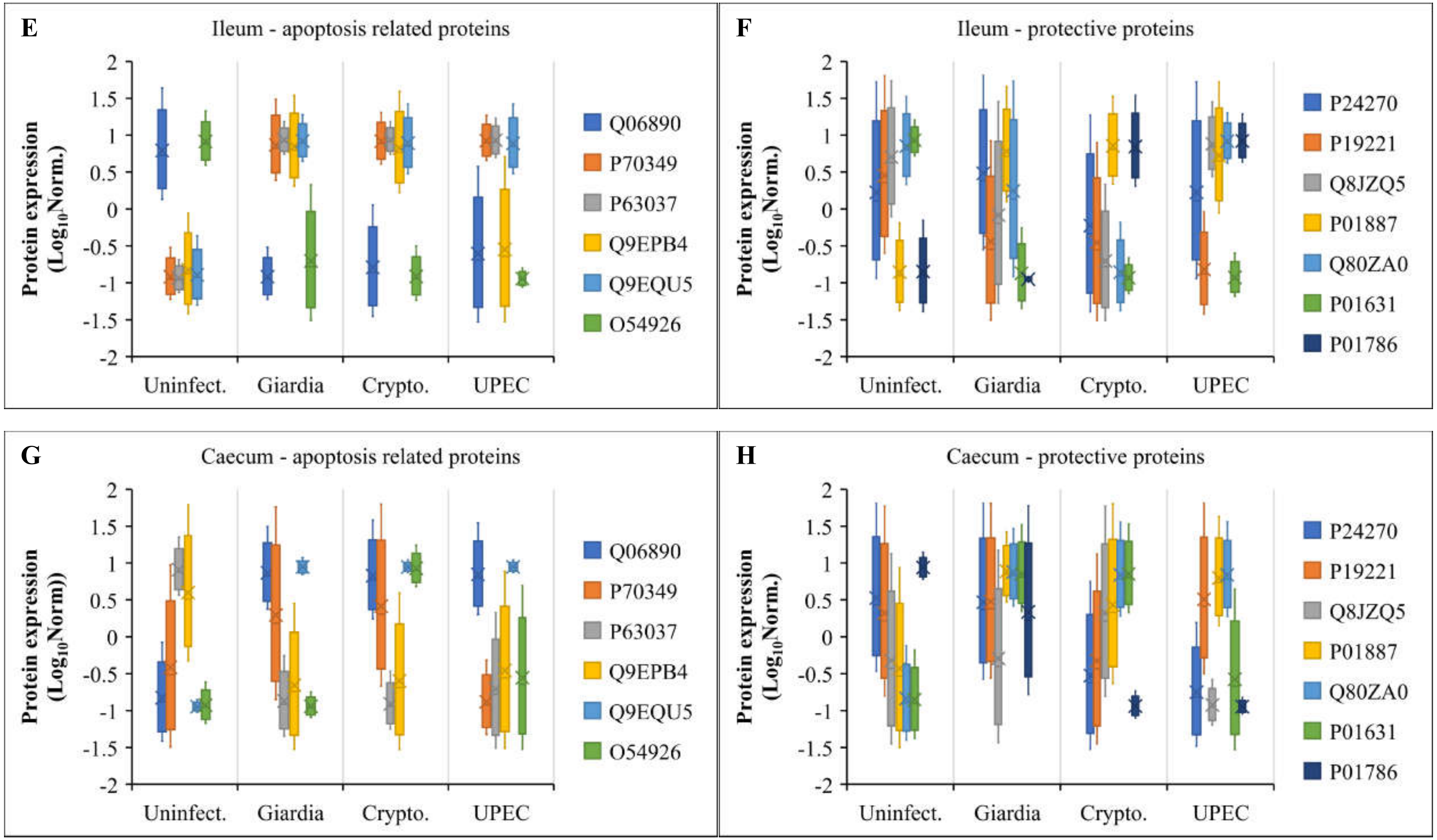
E
Ileum - apoptosis related proteins
Protein expression (Log10Norm.)
Uninfect. Giardia Crypto. UPEC
Q06890 P70349 P63037 Q9EPB4 Q9EQU5 O54926
F
Ileum - protective proteins
Protein expression (Log10Norm.)
Uninfect. Giardia Crypto. UPEC
P24270 P19221 Q8JZQ5 P01887 Q80ZA0 P01631 P01786
G
Caecum - apoptosis related proteins
Protein expression (Log10Norm))
Uninfect. Giardia Crypto. UPEC
Q06890 P70349 P63037 Q9EPB4 Q9EQU5 O54926
H
Caecum - protective proteins
Protein expression (Log10Norm.)
Uninfect. Giardia Crypto. UPEC
P24270 P19221 Q8JZQ5 P01887 Q80ZA0 P01631 P01786

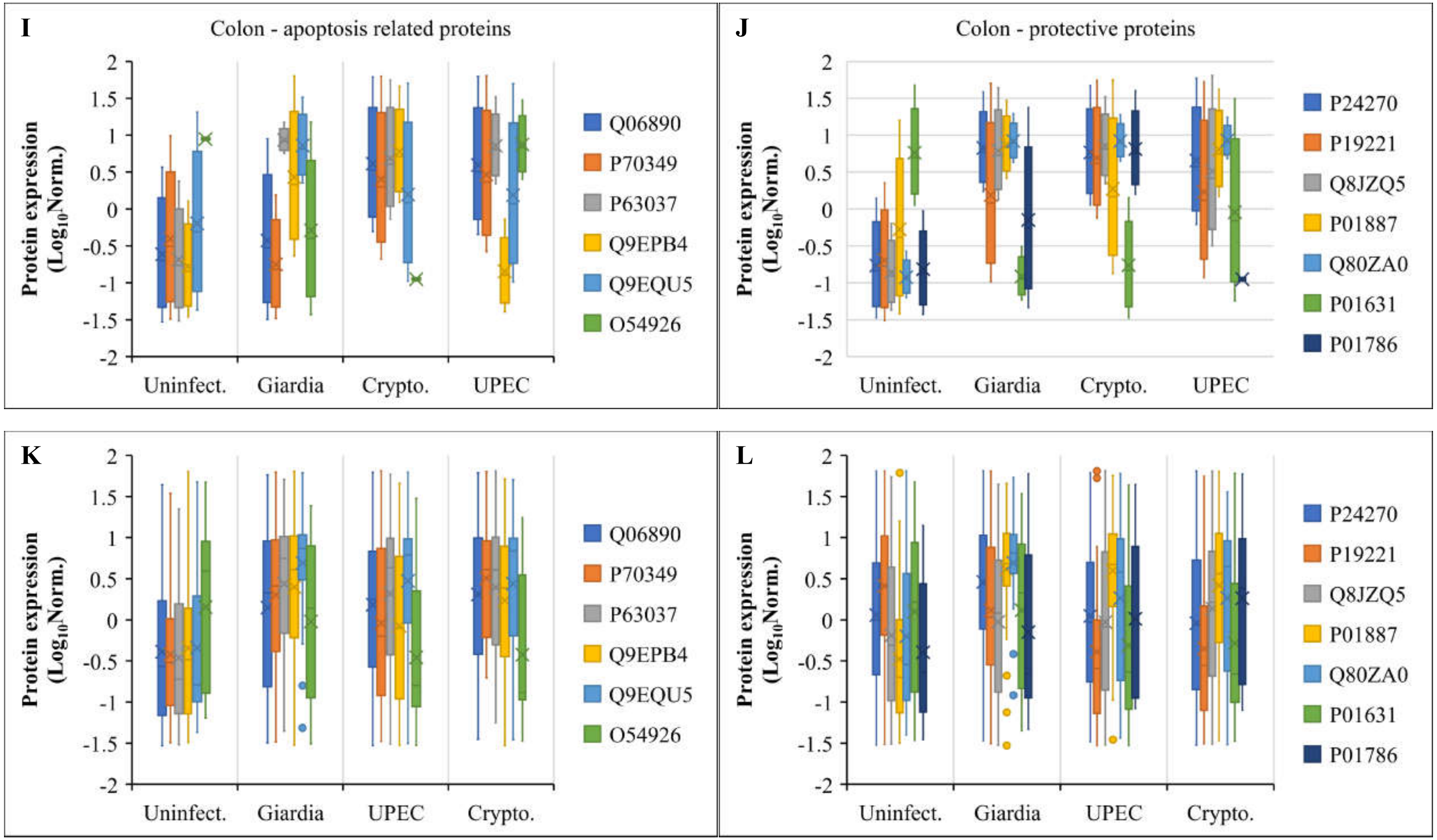

Figure S11. Log_{10}normalised expression of proteins involved in apoptosis process and cellular protection during various infections in the mouse gut sections of (A and B) Duodenum, (C and D) Jejunum, (E and F) Ileum, (G and H) Caecum and (I and J) Colon. Furthermore, an overall inter-species comparison is shown in (K) and (L).

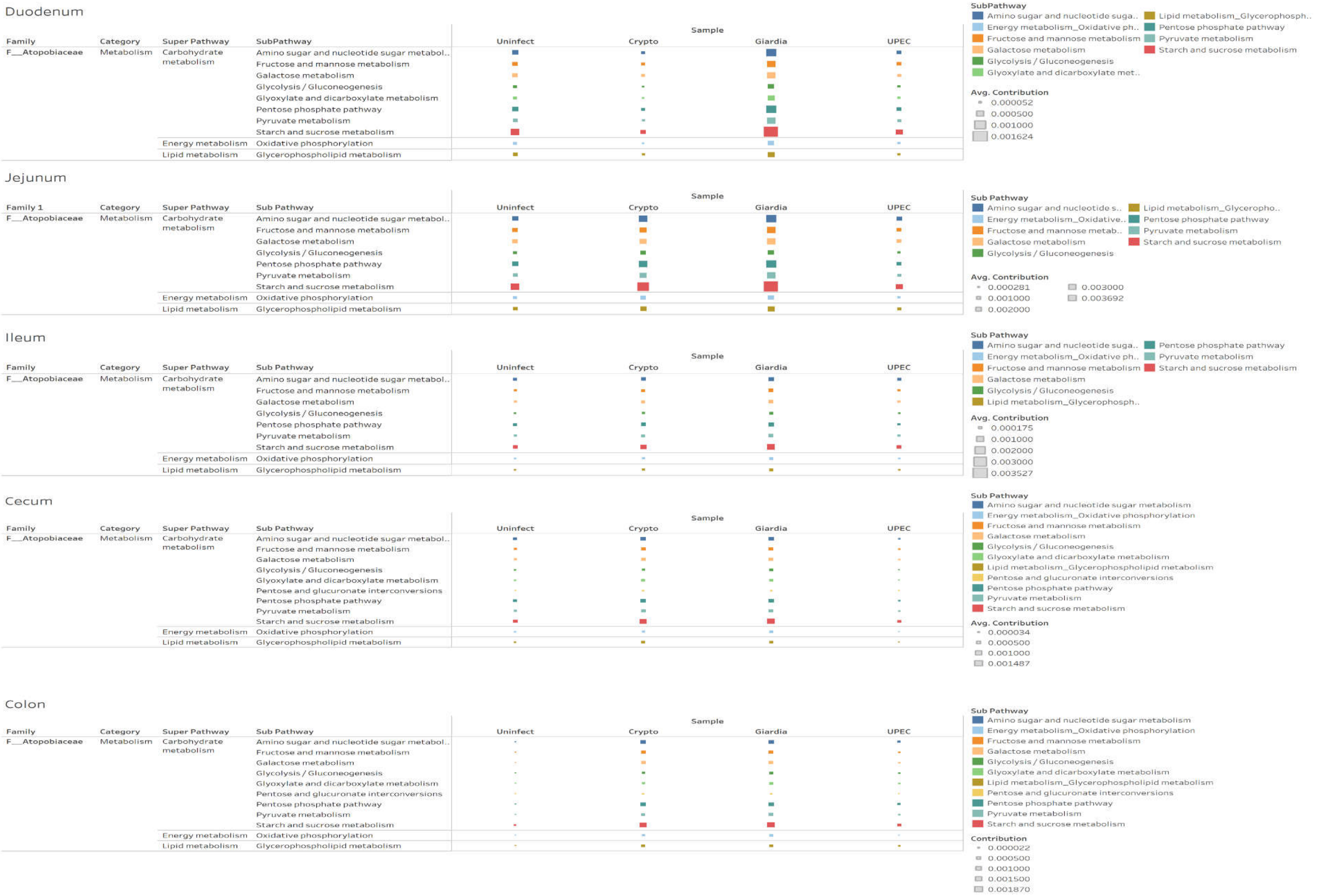
Duodenum
Family
Category
Super Pathway
SubPathway
Sample
Uninfect
Crypto
Giardia
UPEC
F__Atopobiaceae
Metabolism
Carbohydrate metabolism
Amino sugar and nucleotide sugar metabol..
Fructose and mannose metabolism
Galactose metabolism
Glycolysis / Gluconeogenesis
Glyoxylate and dicarboxylate metabolism
Pentose phosphate pathway
Pyruvate metabolism
Starch and sucrose metabolism
Energy metabolism
Oxidative phosphorylation
Lipid metabolism
Glycerophospholipid metabolism
SubPathway
Amino sugar and nucleotide suga..
Energy metabolism_Oxidative ph..
Fructose and mannose metabolism
Galactose metabolism
Glycolysis / Gluconeogenesis
Glyoxylate and dicarboxylate met..
Lipid metabolism_Glycerophosph..
Pentose phosphate pathway
Pyruvate metabolism
Starch and sucrose metabolism
Avg. Contribution
0.000052
0.000500
0.001000
0.001624
Jejunum
Family 1
Category
Super Pathway
Sub Pathway
Sample
Uninfect
Crypto
Giardia
UPEC
F__Atopobiaceae
Metabolism
Carbohydrate metabolism
Amino sugar and nucleotide sugar metabol..
Fructose and mannose metabolism
Galactose metabolism
Glycolysis / Gluconeogenesis
Pentose phosphate pathway
Pyruvate metabolism
Starch and sucrose metabolism
Energy metabolism
Oxidative phosphorylation
Lipid metabolism
Glycerophospholipid metabolism
Sub Pathway
Amino sugar and nucleotide s..
Energy metabolism_Oxidative..
Fructose and mannose metab..
Galactose metabolism
Glycolysis / Gluconeogenesis
Lipid metabolism_Glyceropho..
Pentose phosphate pathway
Pyruvate metabolism
Starch and sucrose metabolism
Avg. Contribution
0.000281
0.001000
0.002000
0.003000
0.003692
Ileum
Family
Category
Super Pathway
Sub Pathway
Sample
Uninfect
Crypto
Giardia
UPEC
F__Atopobiaceae
Metabolism
Carbohydrate metabolism
Amino sugar and nucleotide sugar metabol..
Fructose and mannose metabolism
Galactose metabolism
Glycolysis / Gluconeogenesis
Pentose phosphate pathway
Pyruvate metabolism
Starch and sucrose metabolism
Energy metabolism
Oxidative phosphorylation
Lipid metabolism
Glycerophospholipid metabolism
Sub Pathway
Amino sugar and nucleotide suga..
Energy metabolism_Oxidative ph..
Fructose and mannose metabolism
Galactose metabolism
Glycolysis / Gluconeogenesis
Lipid metabolism_Glycerophosph..
Pentose phosphate pathway
Pyruvate metabolism
Starch and sucrose metabolism
Avg. Contribution
0.000175
0.001000
0.002000
0.003000
0.003527
Cecum
Family
Category
Super Pathway
Sub Pathway
Sample
Uninfect
Crypto
Giardia
UPEC
F__Atopobiaceae
Metabolism
Carbohydrate metabolism
Amino sugar and nucleotide sugar metabol..
Fructose and mannose metabolism
Galactose metabolism
Glycolysis / Gluconeogenesis
Glyoxylate and dicarboxylate metabolism
Pentose and glucuronate interconversions
Pentose phosphate pathway
Pyruvate metabolism
Starch and sucrose metabolism
Energy metabolism
Oxidative phosphorylation
Lipid metabolism
Glycerophospholipid metabolism
Sub Pathway
Amino sugar and nucleotide sugar metabolism
Energy metabolism_Oxidative phosphorylation
Fructose and mannose metabolism
Galactose metabolism
Glycolysis / Gluconeogenesis
Glyoxylate and dicarboxylate metabolism
Lipid metabolism_Glycerophospholipid metabolism
Pentose and glucuronate interconversions
Pentose phosphate pathway
Pyruvate metabolism
Starch and sucrose metabolism
Avg. Contribution
0.000034
0.000500
0.001000
0.001487
Colon
Family
Category
Super Pathway
Sub Pathway
Sample
Uninfect
Crypto
Giardia
UPEC
F__Atopobiaceae
Metabolism
Carbohydrate metabolism
Amino sugar and nucleotide sugar metabol..
Fructose and mannose metabolism
Galactose metabolism
Glycolysis / Gluconeogenesis
Glyoxylate and dicarboxylate metabolism
Pentose and glucuronate interconversions
Pentose phosphate pathway
Pyruvate metabolism
Starch and sucrose metabolism
Energy metabolism
Oxidative phosphorylation
Lipid metabolism
Glycerophospholipid metabolism
Sub Pathway
Amino sugar and nucleotide sugar metabolism
Energy metabolism_Oxidative phosphorylation
Fructose and mannose metabolism
Galactose metabolism
Glycolysis / Gluconeogenesis
Glyoxylate and dicarboxylate metabolism
Lipid metabolism_Glycerophospholipid metabolism
Pentose and glucuronate interconversions
Pentose phosphate pathway
Pyruvate metabolism
Starch and sucrose metabolism
Contribution
0.000022
0.000500
0.001000
0.001500
0.001870

Figure S12. Functional analysis for *Atopobiaceae* family to predict its effect on **carbohydrate metabolism, energy metabolism and lipid metabolism** during cryptosporidiosis and giardiasis.

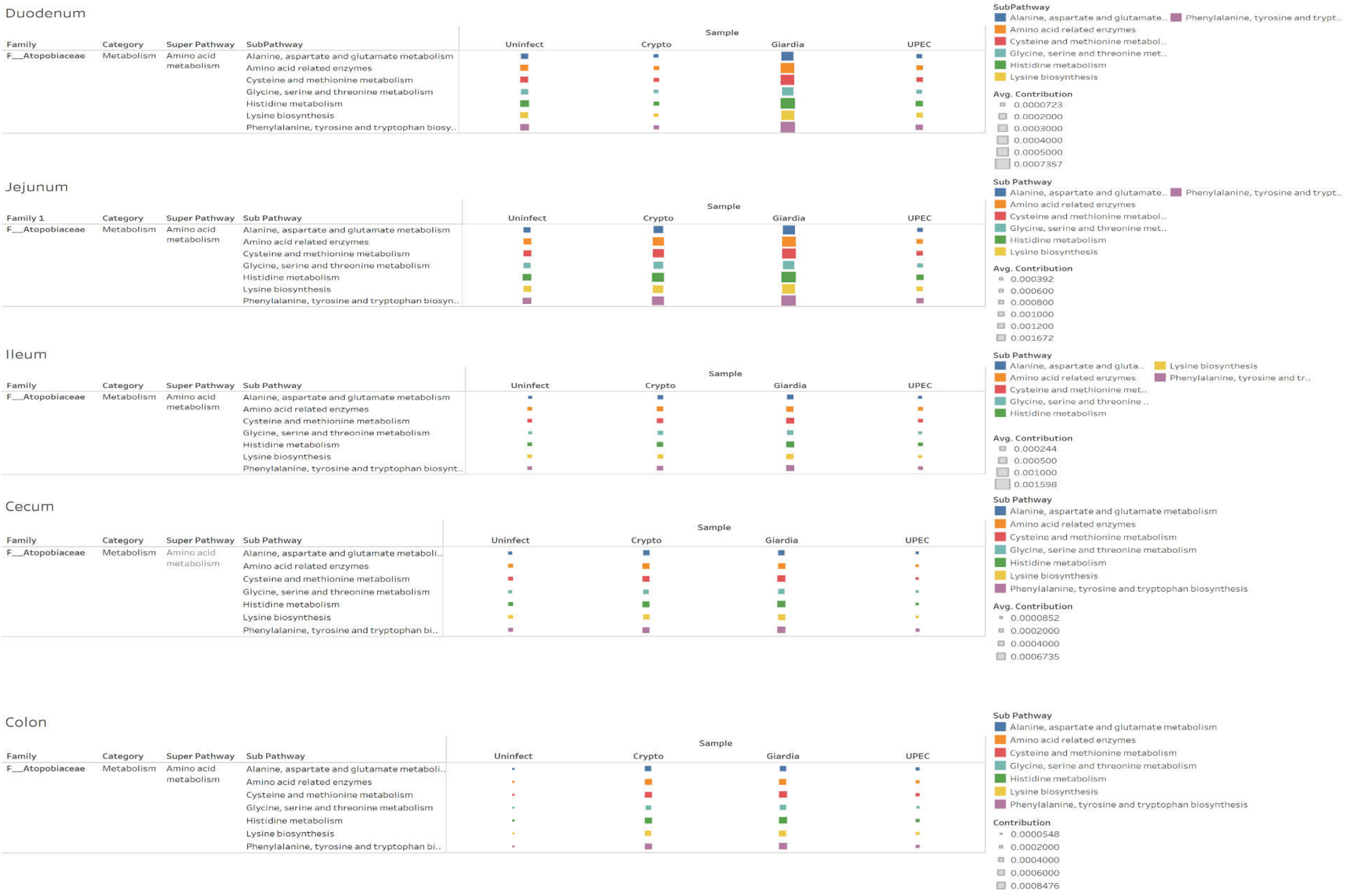
Duodenum
Family
Category
Super Pathway
SubPathway
Sample
Uninfect
Crypto
Giardia
UPEC
F__Atopobiaceae
Metabolism
Amino acid metabolism
Alanine, aspartate and glutamate metabolism
Amino acid related enzymes
Cysteine and methionine metabolism
Glycine, serine and threonine metabolism
Histidine metabolism
Lysine biosynthesis
Phenylalanine, tyrosine and tryptophan biosy..
SubPathway
Alanine, aspartate and glutamate..
Phenylalanine, tyrosine and trypt..
Amino acid related enzymes
Cysteine and methionine metabol..
Glycine, serine and threonine met..
Histidine metabolism
Lysine biosynthesis
Avg. Contribution
0.0000723
0.0002000
0.0003000
0.0004000
0.0005000
0.0007357
Jejunum
Family 1
Category
Super Pathway
Sub Pathway
Sample
Uninfect
Crypto
Giardia
UPEC
F__Atopobiaceae
Metabolism
Amino acid metabolism
Alanine, aspartate and glutamate metabolism
Amino acid related enzymes
Cysteine and methionine metabolism
Glycine, serine and threonine metabolism
Histidine metabolism
Lysine biosynthesis
Phenylalanine, tyrosine and tryptophan biosyn..
Sub Pathway
Alanine, aspartate and glutamate..
Phenylalanine, tyrosine and trypt..
Amino acid related enzymes
Cysteine and methionine metabol..
Glycine, serine and threonine met..
Histidine metabolism
Lysine biosynthesis
Avg. Contribution
0.000392
0.000600
0.000800
0.001000
0.001200
0.001672
Ileum
Family
Category
Super Pathway
Sub Pathway
Sample
Uninfect
Crypto
Giardia
UPEC
F__Atopobiaceae
Metabolism
Amino acid metabolism
Alanine, aspartate and glutamate metabolism
Amino acid related enzymes
Cysteine and methionine metabolism
Glycine, serine and threonine metabolism
Histidine metabolism
Lysine biosynthesis
Phenylalanine, tyrosine and tryptophan biosynt..
Sub Pathway
Alanine, aspartate and gluta..
Lysine biosynthesis
Amino acid related enzymes
Phenylalanine, tyrosine and tr..
Cysteine and methionine met..
Glycine, serine and threonine ..
Histidine metabolism
Avg. Contribution
0.000244
0.000500
0.001000
0.001598
Cecum
Family
Category
Super Pathway
Sub Pathway
Sample
Uninfect
Crypto
Giardia
UPEC
F__Atopobiaceae
Metabolism
Amino acid metabolism
Alanine, aspartate and glutamate metaboli..
Amino acid related enzymes
Cysteine and methionine metabolism
Glycine, serine and threonine metabolism
Histidine metabolism
Lysine biosynthesis
Phenylalanine, tyrosine and tryptophan bi..
Sub Pathway
Alanine, aspartate and glutamate metabolism
Amino acid related enzymes
Cysteine and methionine metabolism
Glycine, serine and threonine metabolism
Histidine metabolism
Lysine biosynthesis
Phenylalanine, tyrosine and tryptophan biosynthesis
Avg. Contribution
0.0000852
0.0002000
0.0004000
0.0006735
Colon
Family
Category
Super Pathway
Sub Pathway
Sample
Uninfect
Crypto
Giardia
UPEC
F__Atopobiaceae
Metabolism
Amino acid metabolism
Alanine, aspartate and glutamate metaboli..
Amino acid related enzymes
Cysteine and methionine metabolism
Glycine, serine and threonine metabolism
Histidine metabolism
Lysine biosynthesis
Phenylalanine, tyrosine and tryptophan bi..
Sub Pathway
Alanine, aspartate and glutamate metabolism
Amino acid related enzymes
Cysteine and methionine metabolism
Glycine, serine and threonine metabolism
Histidine metabolism
Lysine biosynthesis
Phenylalanine, tyrosine and tryptophan biosynthesis
Contribution
0.0000548
0.0002000
0.0004000
0.0006000
0.0008476

Figure S13. – Functional analysis for *Atopobiaceae* family to predict its effect on **amino acid metabolism** during cryptosporidiosis and giardiasis.

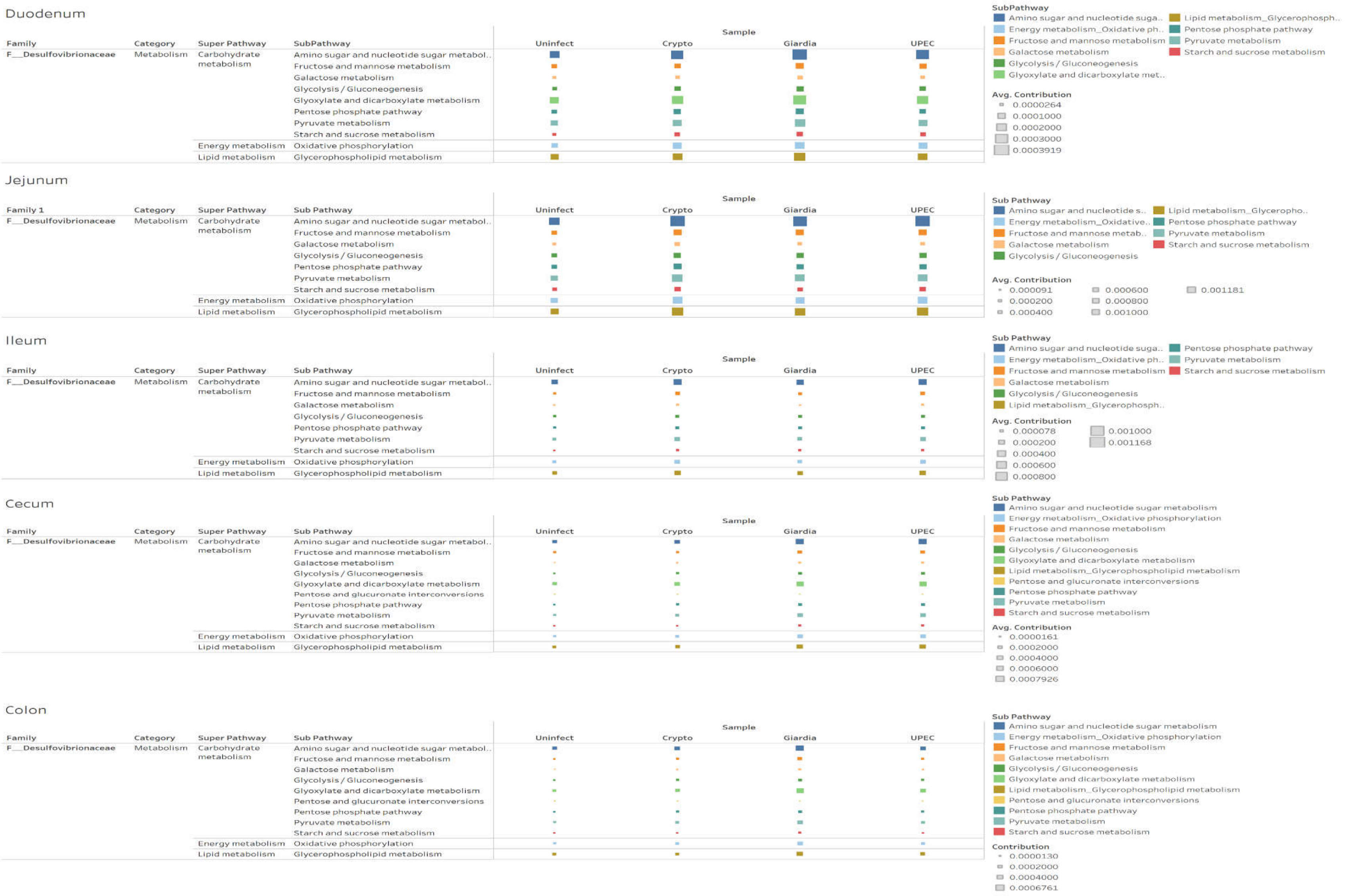
Duodenum
Family
Category
Super Pathway
SubPathway
Sample
Uninfect
Crypto
Giardia
UPEC
F__Desulfovibrionaceae
Metabolism
Carbohydrate metabolism
Amino sugar and nucleotide sugar metabol..
Fructose and mannose metabolism
Galactose metabolism
Glycolysis / Gluconeogenesis
Glyoxylate and dicarboxylate metabolism
Pentose phosphate pathway
Pyruvate metabolism
Starch and sucrose metabolism
Energy metabolism
Oxidative phosphorylation
Lipid metabolism
Glycerophospholipid metabolism
SubPathway
Amino sugar and nucleotide suga..
Energy metabolism_Oxidative ph..
Fructose and mannose metabolism
Galactose metabolism
Glycolysis / Gluconeogenesis
Glyoxylate and dicarboxylate met..
Lipid metabolism_Glycerophosph..
Pentose phosphate pathway
Pyruvate metabolism
Starch and sucrose metabolism
Avg. Contribution
0.0000264
0.0001000
0.0002000
0.0003000
0.0003919
Jejunum
Family 1
Category
Super Pathway
Sub Pathway
Sample
Uninfect
Crypto
Giardia
UPEC
F__Desulfovibrionaceae
Metabolism
Carbohydrate metabolism
Amino sugar and nucleotide sugar metabol..
Fructose and mannose metabolism
Galactose metabolism
Glycolysis / Gluconeogenesis
Pentose phosphate pathway
Pyruvate metabolism
Starch and sucrose metabolism
Energy metabolism
Oxidative phosphorylation
Lipid metabolism
Glycerophospholipid metabolism
Sub Pathway
Amino sugar and nucleotide s..
Energy metabolism_Oxidative..
Fructose and mannose metab..
Galactose metabolism
Glycolysis / Gluconeogenesis
Lipid metabolism_Glyceropho..
Pentose phosphate pathway
Pyruvate metabolism
Starch and sucrose metabolism
Avg. Contribution
0.000091
0.000200
0.000400
0.000600
0.000800
0.001000
0.001181
Ileum
Family
Category
Super Pathway
Sub Pathway
Sample
Uninfect
Crypto
Giardia
UPEC
F__Desulfovibrionaceae
Metabolism
Carbohydrate metabolism
Amino sugar and nucleotide sugar metabol..
Fructose and mannose metabolism
Galactose metabolism
Glycolysis / Gluconeogenesis
Pentose phosphate pathway
Pyruvate metabolism
Starch and sucrose metabolism
Energy metabolism
Oxidative phosphorylation
Lipid metabolism
Glycerophospholipid metabolism
Sub Pathway
Amino sugar and nucleotide suga..
Energy metabolism_Oxidative ph..
Fructose and mannose metabolism
Galactose metabolism
Glycolysis / Gluconeogenesis
Lipid metabolism_Glycerophosph..
Pentose phosphate pathway
Pyruvate metabolism
Starch and sucrose metabolism
Avg. Contribution
0.000078
0.000200
0.000400
0.000600
0.000800
0.001000
0.001168
Cecum
Family
Category
Super Pathway
Sub Pathway
Sample
Uninfect
Crypto
Giardia
UPEC
F__Desulfovibrionaceae
Metabolism
Carbohydrate metabolism
Amino sugar and nucleotide sugar metabol..
Fructose and mannose metabolism
Galactose metabolism
Glycolysis / Gluconeogenesis
Glyoxylate and dicarboxylate metabolism
Pentose and glucuronate interconversions
Pentose phosphate pathway
Pyruvate metabolism
Starch and sucrose metabolism
Energy metabolism
Oxidative phosphorylation
Lipid metabolism
Glycerophospholipid metabolism
Sub Pathway
Amino sugar and nucleotide sugar metabolism
Energy metabolism_Oxidative phosphorylation
Fructose and mannose metabolism
Galactose metabolism
Glycolysis / Gluconeogenesis
Glyoxylate and dicarboxylate metabolism
Lipid metabolism_Glycerophospholipid metabolism
Pentose and glucuronate interconversions
Pentose phosphate pathway
Pyruvate metabolism
Starch and sucrose metabolism
Avg. Contribution
0.0000161
0.0002000
0.0004000
0.0006000
0.0007926
Colon
Family
Category
Super Pathway
Sub Pathway
Sample
Uninfect
Crypto
Giardia
UPEC
F__Desulfovibrionaceae
Metabolism
Carbohydrate metabolism
Amino sugar and nucleotide sugar metabol..
Fructose and mannose metabolism
Galactose metabolism
Glycolysis / Gluconeogenesis
Glyoxylate and dicarboxylate metabolism
Pentose and glucuronate interconversions
Pentose phosphate pathway
Pyruvate metabolism
Starch and sucrose metabolism
Energy metabolism
Oxidative phosphorylation
Lipid metabolism
Glycerophospholipid metabolism
Sub Pathway
Amino sugar and nucleotide sugar metabolism
Energy metabolism_Oxidative phosphorylation
Fructose and mannose metabolism
Galactose metabolism
Glycolysis / Gluconeogenesis
Glyoxylate and dicarboxylate metabolism
Lipid metabolism_Glycerophospholipid metabolism
Pentose and glucuronate interconversions
Pentose phosphate pathway
Pyruvate metabolism
Starch and sucrose metabolism
Contribution
0.0000130
0.0002000
0.0004000
0.0006761

Figure S14. Functional analysis for *Desulfovibrionaceae* family to predict its effect on **carbohydrate metabolism, energy metabolism and lipid metabolism** during cryptosporidiosis and giardiasis.

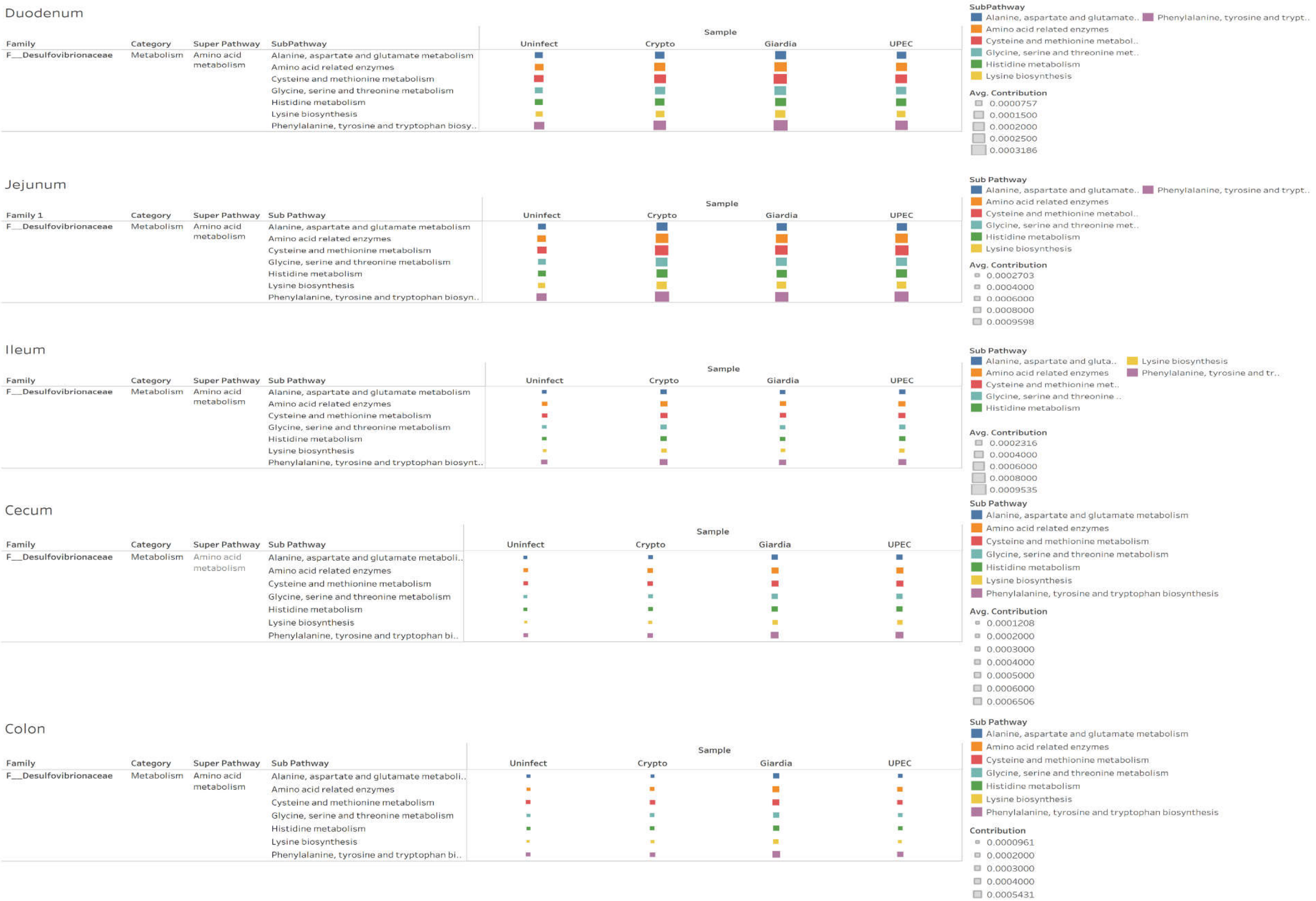
Duodenum
Family
Category
Super Pathway
SubPathway
Sample
Uninfect
Crypto
Giardia
UPEC
F__Desulfovibrionaceae
Metabolism
Amino acid metabolism
Alanine, aspartate and glutamate metabolism
Amino acid related enzymes
Cysteine and methionine metabolism
Glycine, serine and threonine metabolism
Histidine metabolism
Lysine biosynthesis
Phenylalanine, tyrosine and tryptophan biosy..
SubPathway
Alanine, aspartate and glutamate..
Phenylalanine, tyrosine and trypt..
Amino acid related enzymes
Cysteine and methionine metabol..
Glycine, serine and threonine met..
Histidine metabolism
Lysine biosynthesis
Avg. Contribution
0.0000757
0.0001500
0.0002000
0.0002500
0.0003186
Jejunum
Family 1
Category
Super Pathway
Sub Pathway
Sample
Uninfect
Crypto
Giardia
UPEC
F__Desulfovibrionaceae
Metabolism
Amino acid metabolism
Alanine, aspartate and glutamate metabolism
Amino acid related enzymes
Cysteine and methionine metabolism
Glycine, serine and threonine metabolism
Histidine metabolism
Lysine biosynthesis
Phenylalanine, tyrosine and tryptophan biosyn..
Sub Pathway
Alanine, aspartate and glutamate..
Phenylalanine, tyrosine and trypt..
Amino acid related enzymes
Cysteine and methionine metabol..
Glycine, serine and threonine met..
Histidine metabolism
Lysine biosynthesis
Avg. Contribution
0.0002703
0.0004000
0.0006000
0.0008000
0.0009598
Ileum
Family
Category
Super Pathway
Sub Pathway
Sample
Uninfect
Crypto
Giardia
UPEC
F__Desulfovibrionaceae
Metabolism
Amino acid metabolism
Alanine, aspartate and glutamate metabolism
Amino acid related enzymes
Cysteine and methionine metabolism
Glycine, serine and threonine metabolism
Histidine metabolism
Lysine biosynthesis
Phenylalanine, tyrosine and tryptophan biosynt..
Sub Pathway
Alanine, aspartate and gluta..
Lysine biosynthesis
Amino acid related enzymes
Phenylalanine, tyrosine and tr..
Cysteine and methionine met..
Glycine, serine and threonine ..
Histidine metabolism
Avg. Contribution
0.0002316
0.0004000
0.0006000
0.0008000
0.0009535
Cecum
Family
Category
Super Pathway
Sub Pathway
Sample
Uninfect
Crypto
Giardia
UPEC
F__Desulfovibrionaceae
Metabolism
Amino acid metabolism
Alanine, aspartate and glutamate metaboli..
Amino acid related enzymes
Cysteine and methionine metabolism
Glycine, serine and threonine metabolism
Histidine metabolism
Lysine biosynthesis
Phenylalanine, tyrosine and tryptophan bi..
Sub Pathway
Alanine, aspartate and glutamate metabolism
Amino acid related enzymes
Cysteine and methionine metabolism
Glycine, serine and threonine metabolism
Histidine metabolism
Lysine biosynthesis
Phenylalanine, tyrosine and tryptophan biosynthesis
Avg. Contribution
0.0001208
0.0002000
0.0003000
0.0004000
0.0005000
0.0006000
0.0006506
Colon
Family
Category
Super Pathway
Sub Pathway
Sample
Uninfect
Crypto
Giardia
UPEC
F__Desulfovibrionaceae
Metabolism
Amino acid metabolism
Alanine, aspartate and glutamate metaboli..
Amino acid related enzymes
Cysteine and methionine metabolism
Glycine, serine and threonine metabolism
Histidine metabolism
Lysine biosynthesis
Phenylalanine, tyrosine and tryptophan bi..
Sub Pathway
Alanine, aspartate and glutamate metabolism
Amino acid related enzymes
Cysteine and methionine metabolism
Glycine, serine and threonine metabolism
Histidine metabolism
Lysine biosynthesis
Phenylalanine, tyrosine and tryptophan biosynthesis
Contribution
0.0000961
0.0002000
0.0003000
0.0004000
0.0005431

Figure S15. Functional analysis for *Desulfovibrionaceae* family to predict its effect on **amino acid metabolism** during cryptosporidiosis and giardiasis.

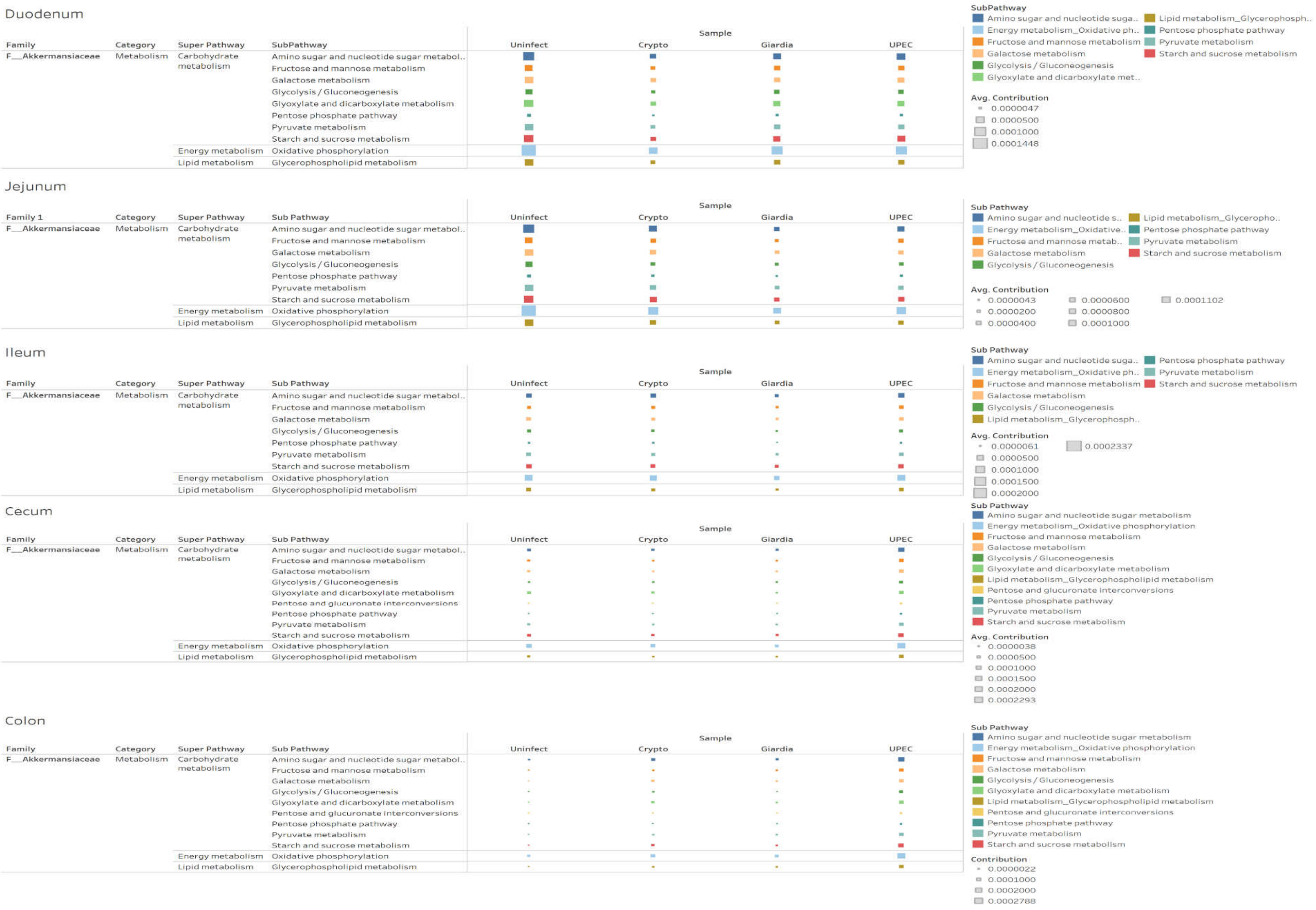

Duodenum
Sample
Family
Category
Super Pathway
SubPathway
Uninfect
Crypto
Giardia
UPEC
F__Akkermansiaceae
Metabolism
Carbohydrate metabolism
Amino sugar and nucleotide sugar metabol..
Fructose and mannose metabolism
Galactose metabolism
Glycolysis / Gluconeogenesis
Glyoxylate and dicarboxylate metabolism
Pentose phosphate pathway
Pyruvate metabolism
Starch and sucrose metabolism
Energy metabolism
Oxidative phosphorylation
Lipid metabolism
Glycerophospholipid metabolism
SubPathway
Amino sugar and nucleotide suga..
Energy metabolism_Oxidative ph..
Fructose and mannose metabolism
Galactose metabolism
Glycolysis / Gluconeogenesis
Glyoxylate and dicarboxylate met..
Lipid metabolism_Glycerophosph..
Pentose phosphate pathway
Pyruvate metabolism
Starch and sucrose metabolism
Avg. Contribution
0.0000047
0.0000500
0.0001000
0.0001448
Jejunum
Sample
Family 1
Category
Super Pathway
Sub Pathway
Uninfect
Crypto
Giardia
UPEC
F__Akkermansiaceae
Metabolism
Carbohydrate metabolism
Amino sugar and nucleotide sugar metabol..
Fructose and mannose metabolism
Galactose metabolism
Glycolysis / Gluconeogenesis
Pentose phosphate pathway
Pyruvate metabolism
Starch and sucrose metabolism
Energy metabolism
Oxidative phosphorylation
Lipid metabolism
Glycerophospholipid metabolism
Sub Pathway
Amino sugar and nucleotide s..
Energy metabolism_Oxidative..
Fructose and mannose metab..
Galactose metabolism
Glycolysis / Gluconeogenesis
Lipid metabolism_Glycerophо..
Pentose phosphate pathway
Pyruvate metabolism
Starch and sucrose metabolism
Avg. Contribution
0.0000043
0.0000200
0.0000400
0.0000600
0.0000800
0.0001000
0.0001102
Ileum
Sample
Family
Category
Super Pathway
Sub Pathway
Uninfect
Crypto
Giardia
UPEC
F__Akkermansiaceae
Metabolism
Carbohydrate metabolism
Amino sugar and nucleotide sugar metabol..
Fructose and mannose metabolism
Galactose metabolism
Glycolysis / Gluconeogenesis
Pentose phosphate pathway
Pyruvate metabolism
Starch and sucrose metabolism
Energy metabolism
Oxidative phosphorylation
Lipid metabolism
Glycerophospholipid metabolism
Sub Pathway
Amino sugar and nucleotide suga..
Energy metabolism_Oxidative ph..
Fructose and mannose metabolism
Galactose metabolism
Glycolysis / Gluconeogenesis
Lipid metabolism_Glycerophosph..
Pentose phosphate pathway
Pyruvate metabolism
Starch and sucrose metabolism
Avg. Contribution
0.0000061
0.0000500
0.0001000
0.0001500
0.0002000
0.0002337
Cecum
Sample
Family
Category
Super Pathway
Sub Pathway
Uninfect
Crypto
Giardia
UPEC
F__Akkermansiaceae
Metabolism
Carbohydrate metabolism
Amino sugar and nucleotide sugar metabol..
Fructose and mannose metabolism
Galactose metabolism
Glycolysis / Gluconeogenesis
Glyoxylate and dicarboxylate metabolism
Pentose and glucuronate interconversions
Pentose phosphate pathway
Pyruvate metabolism
Starch and sucrose metabolism
Energy metabolism
Oxidative phosphorylation
Lipid metabolism
Glycerophospholipid metabolism
Sub Pathway
Amino sugar and nucleotide sugar metabolism
Energy metabolism_Oxidative phosphorylation
Fructose and mannose metabolism
Galactose metabolism
Glycolysis / Gluconeogenesis
Glyoxylate and dicarboxylate metabolism
Lipid metabolism_Glycerophospholipid metabolism
Pentose and glucuronate interconversions
Pentose phosphate pathway
Pyruvate metabolism
Starch and sucrose metabolism
Avg. Contribution
0.0000038
0.0000500
0.0001000
0.0001500
0.0002000
0.0002293
Colon
Sample
Family
Category
Super Pathway
Sub Pathway
Uninfect
Crypto
Giardia
UPEC
F__Akkermansiaceae
Metabolism
Carbohydrate metabolism
Amino sugar and nucleotide sugar metabol..
Fructose and mannose metabolism
Galactose metabolism
Glycolysis / Gluconeogenesis
Glyoxylate and dicarboxylate metabolism
Pentose and glucuronate interconversions
Pentose phosphate pathway
Pyruvate metabolism
Starch and sucrose metabolism
Energy metabolism
Oxidative phosphorylation
Lipid metabolism
Glycerophospholipid metabolism
Sub Pathway
Amino sugar and nucleotide sugar metabolism
Energy metabolism_Oxidative phosphorylation
Fructose and mannose metabolism
Galactose metabolism
Glycolysis / Gluconeogenesis
Glyoxylate and dicarboxylate metabolism
Lipid metabolism_Glycerophospholipid metabolism
Pentose and glucuronate interconversions
Pentose phosphate pathway
Pyruvate metabolism
Starch and sucrose metabolism
Contribution
0.0000022
0.0001000
0.0002000
0.0002788

Figure S16. Functional analysis for *Akkermansiaceae* family to predict its effect on **carbohydrate metabolism, energy metabolism and lipid metabolism** during cryptosporidiosis and giardiasis.

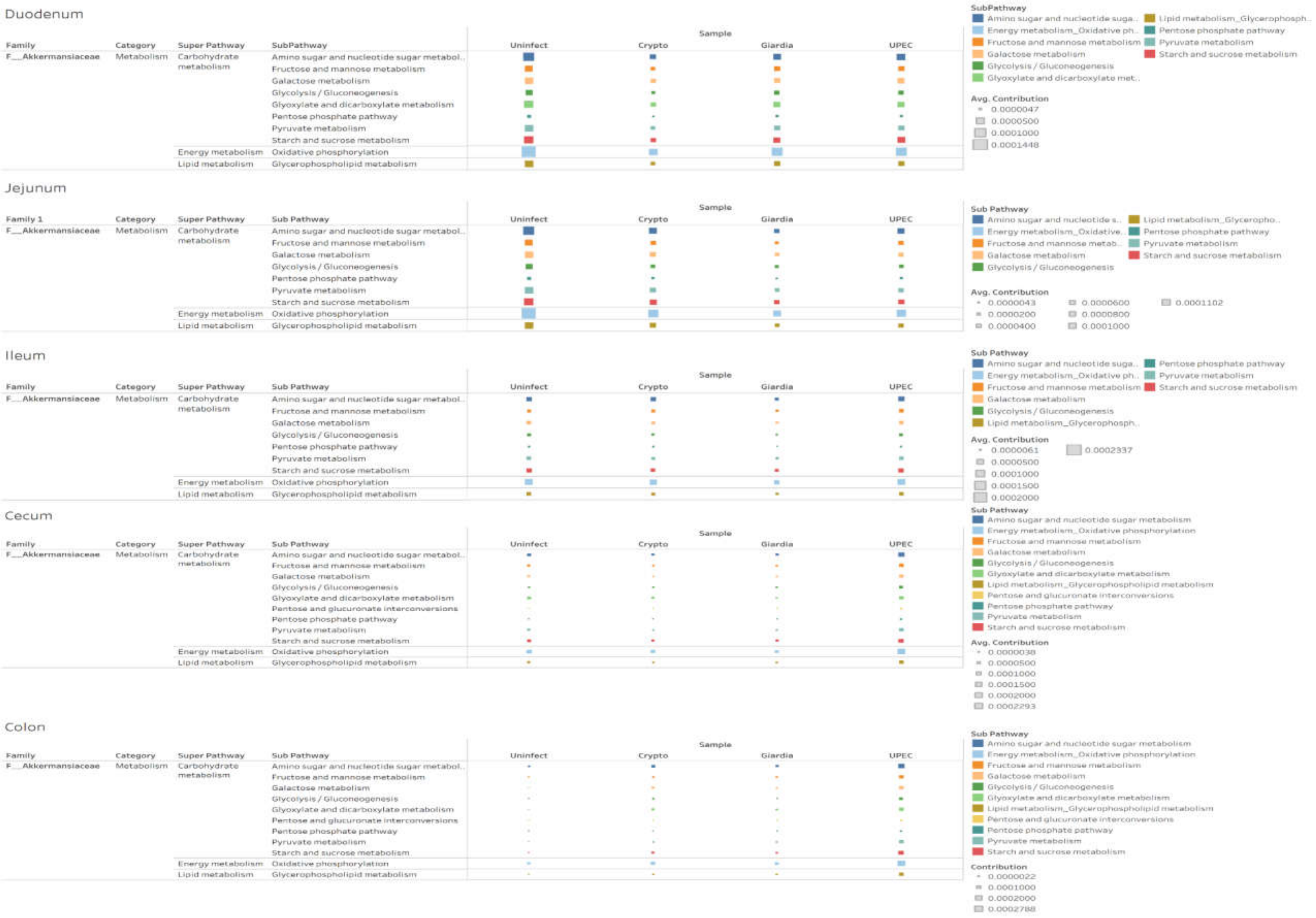
Duodenum
Family
Category
Super Pathway
SubPathway
Sample
Uninfect
Crypto
Giardia
UPEC
F__Akkermansiaceae
Metabolism
Carbohydrate metabolism
Amino sugar and nucleotide sugar metabol..
Fructose and mannose metabolism
Galactose metabolism
Glycolysis / Gluconeogenesis
Glyoxylate and dicarboxylate metabolism
Pentose phosphate pathway
Pyruvate metabolism
Starch and sucrose metabolism
Energy metabolism
Oxidative phosphorylation
Lipid metabolism
Glycerophospholipid metabolism
SubPathway
Amino sugar and nucleotide suga..
Energy metabolism_Oxidative ph..
Fructose and mannose metabolism
Galactose metabolism
Glycolysis / Gluconeogenesis
Glyoxylate and dicarboxylate met..
Lipid metabolism_Glycerophosph..
Pentose phosphate pathway
Pyruvate metabolism
Starch and sucrose metabolism
Avg. Contribution
0.0000047
0.0000500
0.0001000
0.0001448
Jejunum
Family 1
Category
Super Pathway
Sub Pathway
Sample
Uninfect
Crypto
Giardia
UPEC
F__Akkermansiaceae
Metabolism
Carbohydrate metabolism
Amino sugar and nucleotide sugar metabol..
Fructose and mannose metabolism
Galactose metabolism
Glycolysis / Gluconeogenesis
Pentose phosphate pathway
Pyruvate metabolism
Starch and sucrose metabolism
Energy metabolism
Oxidative phosphorylation
Lipid metabolism
Glycerophospholipid metabolism
Sub Pathway
Amino sugar and nucleotide s..
Energy metabolism_Oxidative..
Fructose and mannose metab..
Galactose metabolism
Glycolysis / Gluconeogenesis
Lipid metabolism_Glycerophо..
Pentose phosphate pathway
Pyruvate metabolism
Starch and sucrose metabolism
Avg. Contribution
0.0000043
0.0000200
0.0000400
0.0000600
0.0000800
0.0001000
0.0001102
Ileum
Family
Category
Super Pathway
Sub Pathway
Sample
Uninfect
Crypto
Giardia
UPEC
F__Akkermansiaceae
Metabolism
Carbohydrate metabolism
Amino sugar and nucleotide sugar metabol..
Fructose and mannose metabolism
Galactose metabolism
Glycolysis / Gluconeogenesis
Pentose phosphate pathway
Pyruvate metabolism
Starch and sucrose metabolism
Energy metabolism
Oxidative phosphorylation
Lipid metabolism
Glycerophospholipid metabolism
Sub Pathway
Amino sugar and nucleotide suga..
Energy metabolism_Oxidative ph..
Fructose and mannose metabolism
Galactose metabolism
Glycolysis / Gluconeogenesis
Lipid metabolism_Glycerophosph..
Pentose phosphate pathway
Pyruvate metabolism
Starch and sucrose metabolism
Avg. Contribution
0.0000061
0.0000500
0.0001000
0.0001500
0.0002000
0.0002337
Cecum
Family
Category
Super Pathway
Sub Pathway
Sample
Uninfect
Crypto
Giardia
UPEC
F__Akkermansiaceae
Metabolism
Carbohydrate metabolism
Amino sugar and nucleotide sugar metabol..
Fructose and mannose metabolism
Galactose metabolism
Glycolysis / Gluconeogenesis
Glyoxylate and dicarboxylate metabolism
Pentose and glucuronate interconversions
Pentose phosphate pathway
Pyruvate metabolism
Starch and sucrose metabolism
Energy metabolism
Oxidative phosphorylation
Lipid metabolism
Glycerophospholipid metabolism
Sub Pathway
Amino sugar and nucleotide sugar metabolism
Energy metabolism_Oxidative phosphorylation
Fructose and mannose metabolism
Galactose metabolism
Glycolysis / Gluconeogenesis
Glyoxylate and dicarboxylate metabolism
Lipid metabolism_Glycerophospholipid metabolism
Pentose and glucuronate interconversions
Pentose phosphate pathway
Pyruvate metabolism
Starch and sucrose metabolism
Avg. Contribution
0.0000038
0.0000500
0.0001000
0.0001500
0.0002000
0.0002293
Colon
Family
Category
Super Pathway
Sub Pathway
Sample
Uninfect
Crypto
Giardia
UPEC
F__Akkermansiaceae
Metabolism
Carbohydrate metabolism
Amino sugar and nucleotide sugar metabol..
Fructose and mannose metabolism
Galactose metabolism
Glycolysis / Gluconeogenesis
Glyoxylate and dicarboxylate metabolism
Pentose and glucuronate interconversions
Pentose phosphate pathway
Pyruvate metabolism
Starch and sucrose metabolism
Energy metabolism
Oxidative phosphorylation
Lipid metabolism
Glycerophospholipid metabolism
Sub Pathway
Amino sugar and nucleotide sugar metabolism
Energy metabolism_Oxidative phosphorylation
Fructose and mannose metabolism
Galactose metabolism
Glycolysis / Gluconeogenesis
Glyoxylate and dicarboxylate metabolism
Lipid metabolism_Glycerophospholipid metabolism
Pentose and glucuronate interconversions
Pentose phosphate pathway
Pyruvate metabolism
Starch and sucrose metabolism
Contribution
0.0000022
0.0001000
0.0002000
0.0002788

Figure S 17. Functional analysis for *Akkermansiaceae* family to predict its effect on **amino acid metabolism** during cryptosporidiosis and giardiasis.
